# Characterization of the Inducible and Slow-Releasing Hydrogen Sulfide and Persulfide Donor P*: Insights into Hydrogen Sulfide Signaling

**DOI:** 10.3390/antiox10071049

**Published:** 2021-06-29

**Authors:** Modesta Trummer, Erwan Galardon, Anita Fischer, Stefan Toegel, Bernd Mayer, Guenter Steiner, Burkhard Kloesch

**Affiliations:** 1 Ludwig Boltzmann Institute for Arthritis and Rehabilitation, 1090 Vienna, Austria; modesta.trummer@ar.lbg.ac.at (M.T.); anita.fischer@meduniwien.ac.at (A.F.); stefan.toegel@meduniwien.ac.at (S.T.); guenter.steiner@meduniwien.ac.at (G.S.); 2 Department of Pharmacology and Toxicology, Institute of Pharmaceutical Sciences, Karl-Franzens-University of Graz, 8010 Graz, Austria; bernhard-michael.mayer@uni-graz.at; 3 UMR 8601, LCBPT, CNRS-Université de Paris, 75270 Paris, France; erwan.galardon@parisdescartes.fr; 4 Karl Chiari Lab for Orthopaedic Biology, Department of Orthopedics and Trauma Surgery, Medical University of Vienna, 1090 Vienna, Austria; 5 Division of Rheumatology, Department of Internal Medicine III, Medical University of Vienna, 1090 Vienna, Austria

**Keywords:** hydrogen sulfide, polysulfides, ATDC5, human chondrocytes, osteoarthritis, oxidative stress, heme oxygenase-1, inflammation

## Abstract

Hydrogen sulfide (H_2_S) is an important mediator of inflammatory processes. However, controversial findings also exist, and its underlying molecular mechanisms are largely unknown. Recently, the byproducts of H_2_S, per-/polysulfides, emerged as biological mediators themselves, highlighting the complex chemistry of H_2_S. In this study, we characterized the biological effects of P*, a slow-releasing H_2_S and persulfide donor. To differentiate between H_2_S and polysulfide-derived effects, we decomposed P* into polysulfides. P* was further compared to the commonly used fast-releasing H_2_S donor sodium hydrogen sulfide (NaHS). The effects on oxidative stress and interleukin-6 (IL-6) expression were assessed in ATDC5 cells using superoxide measurement, qPCR, ELISA, and Western blotting. The findings on IL-6 expression were corroborated in primary chondrocytes from osteoarthritis patients. In ATDC5 cells, P* not only induced the expression of the antioxidant enzyme heme oxygenase-1 via per-/polysulfides, but also induced activation of Akt and p38 MAPK. NaHS and P* significantly impaired menadione-induced superoxide production. P* reduced IL-6 levels in both ATDC5 cells and primary chondrocytes dependent on H_2_S release. Taken together, P* provides a valuable research tool for the investigation of H_2_S and per-/polysulfide signaling. These data demonstrate the importance of not only H_2_S, but also per-/polysulfides as bioactive signaling molecules with potent anti-inflammatory and, in particular, antioxidant properties.

## 1. Introduction

Osteoarthritis (OA) is a degenerative joint disease characterized by pain, cartilage destruction, and disability. An imbalance occurs between the synthesis and degradation of the extracellular matrix tissue and involves mechanical, metabolic, and inflammatory factors [1,2]. OA represents the most common form of arthritis, but most disease-modifying OA drugs and therapies have shown little clinical efficacy [3]. Among the non-pharmacological therapies for OA, traditional balneotherapy in hydrogen sulfide (H_2_S)-rich water is still widely used and recommended by the guidelines of the OA Research Society International (OARSI) [3]. In different in vitro and in vivo OA models, H_2_S exerted anti-inflammatory, antioxidant, and pain-relieving effects [4,5,6,7,8]. Yet, the effects of H_2_S on signaling and transcription pathways varied greatly, depending on the cell type as well as the H_2_S donor and the concentrations used [9].

Nowadays, H_2_S is recognized as the third “gasotransmitter” alongside nitric oxide and carbon monoxide [10]. These molecules are gases at standard temperature and pressure but are solutes when acting as physiological signaling molecules [11]. H_2_S is synthesized endogenously in most tissues, mainly from l-cysteine by cystathionine-γ-lyase and cystathionine β-synthase [12,13]. Mustafa et al. [14] first described the key molecular mechanism of H_2_S signaling, the so-called S-sulfhydration. In this oxidative posttranslational modification, H_2_S can promote the conversion of reactive cysteine residues into their respective hydropersulfides (-SSH), which are more reactive [15]. A well-known target of S-sulfhydration is Kelch-like ECH-associated protein 1 (Keap1), the negative regulator of the transcription factor nuclear factor erythroid 2-related factor 2 (Nrf2). Calvert et al. [16] first demonstrated that H_2_S induced the nuclear accumulation of Nrf2. Shortly thereafter, H_2_S was described to activate Nrf2 via the S-sulfhydration of Keap1 at C151 [17,18] or between C226 and C613 [19]. Through Nrf2 induction, H_2_S can coordinately enhance the expression of a series of Nrf2-regulated cytoprotective and antioxidant genes, e.g., heme oxygenase-1 (HO-1) and NADPH quinone dehydrogenase 1 (NQO1). HO-1 is the rate-limiting enzyme in heme catabolism and plays a protective role against numerous diseases, including inflammation, diabetes, cardiovascular diseases, obesity, neurodegenerative disorders, and OA [20,21,22,23,24,25,26,27]. However, recent evidence indicates that S-sulfhydration is more likely attributed to persulfides and polysulfides than H_2_S [28,29,30,31]. Congruently, polysulfides as well as the organosulfur compound diallyl trisulfide were also demonstrated to induce Nrf2 through the S-sulfhydration of Keap1 [32,33,34]. Sulfane sulfur species can be generated from H_2_S through enzymatic and non-enzymatic pathways [35], highlighting the complex chemistry of the various sulfur derivates in a biological context.

**Figure 1:**
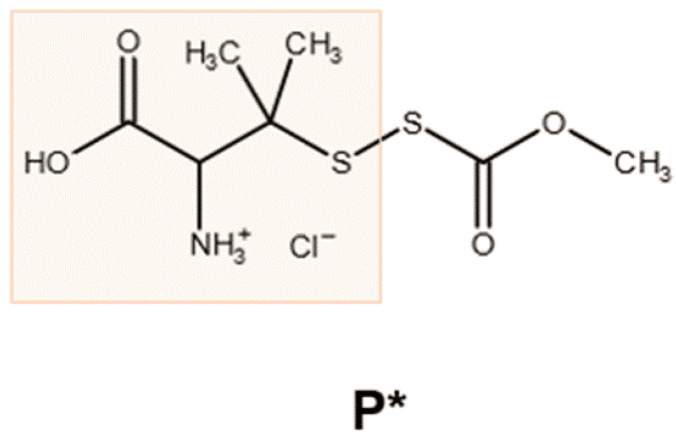
Chemical structure of P*. The reaction of penicillamine (orange box) with methoxycarbonylsulfenyl chloride generates the ammonium salt P*, which decomposes in the presence of thiols, leading to the generation of H_2_S and polysulfides [38].

Sodium hydrogen sulfide (NaHS) and disodium sulfide, both of which generate H_2_S rapidly, serve as the most commonly used experimental H_2_S donors. Recently, several slow-releasing and inducible H_2_S donors have been synthesized [36,37]. Among them, P* (Figure 1) was described in 2014 by Artaud and Galardon [38]. P* is chemically derived from penicillamine and offers several beneficial properties: In aqueous buffers, it generates a metastable persulfide and a mixture of polysulfides as end-products, which can inhibit caspase-3 activity [39]. Moreover, P* also releases H_2_S, but only in the presence of thiols such as cysteine or glutathione.

As little is known about the biological effects of this compound, we studied the cellular response to P* administration in the context of OA pathobiology. To examine the potential of P* in vitro as a slow and inducible H_2_S and persulfide donor in comparison to NaHS, we determined the effect of both substances on oxidative stress in the murine chondrocyte-like cell line ATDC5. To assess the anti-inflammatory capacity of P*, we determined IL-6 expression in ATDC5 cells and human chondrocytes derived from OA patients. Finally, we attempted to elucidate the pathway underlying P* signaling.

## 2. Materials and Methods

### 2.1. Materials

P* was synthesized as previously reported [38]. Stock solutions of NaHS and P* were prepared in H_2_O. To decompose P*, P* was diluted in cell culture medium in the required concentrations in the presence of the equimolar concentrations of l-cysteine and was left uncapped overnight at 37 °C to allow for the evaporation of H_2_S. EDTA, l-cysteine, Igepal CA-630, NaHS, *N*-acetyl cysteine (NAC), neocuproine, and RIPA buffer were purchased from Sigma-Aldrich (Vienna, Austria). Complete Protease Inhibitor^TM^ Cocktail and PhosSTOP^TM^ Phosphatase Inhibitor Cocktail were purchased from Roche Life Science (Vienna, Austria). Hydroethidine was obtained from Calbiochem (Merck4Biosciences, Darmstadt, Germany). All antibodies were purchased from Cell Signaling Technology Europe (Leiden, The Netherlands). The detailed antibody list is readily provided by the authors upon request. If not otherwise stated, all other substances were obtained from MedChemExpress (THP Medical Products, Vienna, Austria).

### 2.2. Cell Culture

The murine chondrogenic cell line ATDC5 (ECACC 99072806) was obtained from the European Collection of Authenticated Cell Cultures, Salisbury, UK. The cells were maintained in Dulbecco’s Modified Eagle’s Medium/Ham’s Nutrient Mixture F12 (DMEM/F12) supplemented with 10% heat-inactivated fetal bovine serum (FBS), 50 U/mL penicillin, and 50 µg/mL streptomycin (Gibco Life Technologies, Thermo Fisher Scientific, Inc. Waltham, Vienna, Austria). Cells were maintained in a 37 °C humidified incubator with 5% CO_2_. To induce IL-6 expression, cells were stimulated for 24 h with recombinant murine IL-1β (10 ng/mL) and IFN-γ (100 ng/mL), (Peprotech, London, UK).

Human osteoarthritic chondrocytes were isolated from femoral condyles and tibial plateaus of articular cartilage as described previously [40], with written informed consent and in accordance with the terms of the ethics committee of the Medical University of Vienna (EK-No. 1822/2017). The isolated cells were maintained in Dulbecco’s Modified Eagle’s Medium + GlutaMAX (Gibco Life Technologies) containing 4.5 g/L D-Glucose supplemented with 10% FBS, 100 U/mL penicillin, 100 µg/mL streptomycin, and 0.25 µg/mL Amphotericin B, (Gibco Life Technologies) at 37 °C. For all assays, primary chondrocytes were used at passage 0 to preserve the chondrocyte phenotype. To induce IL-6 or MMP-13 mRNA expression, cells were stimulated with recombinant human IL-1β (10 ng/mL) for 24 h.

### 2.3. H_2_S Fluorescence Measurement

We monitored the H_2_S release and intracellular uptake from P* with the H_2_S fluorescent probe 3-oxo-3H-spiro-[isobenzofuran-1,9’-xanthene]-3’,6’-diyl-*bis*(2-(pyridin-2-yl-di-sulfanyl)-benzoate), (WSP-5) in the ATDC5 cells. The ATDC5 cells were grown on glass coverslips in a 12-well plate (8 × 10^4^ cells/well) for 24 h. The cells were washed once with FBS-free DMEM/F12 and incubated with 10 µM WSP-5 (in FBS-free DMEM/F12 + 100 µM hexadecyl (trimethyl)ammonium bromide) for 30 min. After washing the cells with phosphate-buffered saline (PBS), they were incubated with 1 mM P* (in FBS-free DMEM/F12 + 100 µM hexadecyl (trimethyl)ammonium bromide) for 30 min. Afterwards, fluorescence imaging was performed at room temperature using a Zeiss Axiovert 200 equipped with a xenon lamp, polychromator, Chroma filters (exciter: ET470/40; emitter: ET525/50; beamsplitter: T495lp), and a CoolSNAP fx-HQ CCD-camera (Visitron Systems GmbH, Puchheim, Germany). For each condition, 8 images were taken randomly and analyzed using ImageJ (https://imagej.nih.gov/ij/, accessed in 2015). For background correction, fluorescence was measured in cell-free areas.

### 2.4. Trypan Blue Staining

The number of viable cells was assessed using trypan blue staining. The ATDC5 cells were grown in 12-well plates (5 × 10^4^ cells/well) for 48 h, followed by 24 h of incubation with or without NaHS or P*. Cells were washed once with PBS and trypsinized. After centrifugation at 500× *g* for 2 min, the cell pellets were resuspended in 1 mL of fresh medium. Cell suspensions were diluted 1:1 in 0.4% trypan blue solution and counted using the DeNovix CellDrop^TM^ Cell Counter (Biozym, Vienna, Austria). For a positive control, the cell suspensions were boiled for 5 min at 95 °C.

### 2.5. MTT Measurement

MTT was used to measure cell viability in isolated primary human chondrocytes. Cells were grown in a 96-well plate (5 × 10^3^ cells/well) until 80% confluency was reached. After 24 h of treatment with NaHS or P*, an MTT assay was performed according to manufacturer protocol (Biomedica, Vienna, Austria).

### 2.6. Western Blot Analysis

ATDC5 cells were seeded in 6-well plates (1 × 10^5^ cells/well) and grown for 48 h. Following different treatments, the cells were washed twice with PBS and directly lysed in 1-fold Laemmli buffer. To minimize viscosity, cell lysates were treated with a short impulse of ultrasound (15 s) and boiled for 5 min at 95 °C afterwards. Equal amounts of protein were run on 10% SDS-PAGE mini gels and electro-transferred to PVDF membranes (Bio-Rad Laboratories Ges.m.b.H., Vienna, Austria). After blocking for 1 h at room temperature with 5% non-fat dried milk in PBS + 0.1% Tween 20, the membranes were incubated with primary antibodies overnight at 4 °C. After 2 h of incubation with horseradish peroxidase coupled anti-rabbit secondary antibody, the proteins were detected using the enhanced chemiluminescence (ECL) WesternBright detection system (Advansta Inc., Bio-Rad Laboratories Ges.m.b.H., Vienna, Austria) and a GeneGnome detection device (Syngene, Cambridge, UK). Relative density was determined with Syngene GeneTool software and adjusted to tubulin or the corresponding unphosphorylated protein as a reference protein.

### 2.7. Preparation of Nuclear-Enriched Fractions

ATDC5 cells were seeded in 6-well plates (1 × 10^5^ cells/well) and grown for 48 h. After 1 h of incubation with 0.5 mM NaHS or 0.5 mM P*, the cells were washed once with PBS and were mechanically scraped off using 0.85 mL of HEN-buffer (pH 7.8) composed of 100 mM 4-(2-hydroxyethyl)-1-piperazineethanesulfonic acid, 1 mM EDTA, 0.1 mM neocuproine, proteinase inhibitors (Complete^®^, Roche Life Science, Vienna, Austria), and phosphatase inhibitors (PhosSTOP^TM^, Roche Life Science, Vienna, Austria). After centrifugation (1000× *g* for 5 min at room temperature), the cells were incubated on ice in 0.85 mL of HEN-buffer supplemented with 0.2% Igepal CA-630 for 3 min. After centrifugation (1000× *g* for 5 min at 4 °C), the supernatants were carefully collected (cytosolic fraction). Pellets (nuclear fraction) were incubated on ice in 15 µL RIPA buffer (#R0278, Sigma-Aldrich, Vienna, Austria) for 10 min. After the addition of 5-fold Laemmli buffer, the cytosolic and nuclear fractions were homogenized by sonication for 10 s and boiled for 5 min at 95 °C afterwards.

### 2.8. Transfection

ATDC5 cells were grown in 12-well plates (4 × 10^4^ cells/well) and transfected the following day with Nrf2 siRNA (SR427248, OriGene Inc., Rockville, MD, USA) or scrambled negative control siRNA (SR30004, OriGene) at a final concentration of 10 nM using the siTran 2.0 transfection reagent (TT320001, OriGene) or Lipofectamine 3000 (Invitrogen, Thermo Fisher Scientific, Vienna, Austria) according to the manufacturer’s instructions. At 6 h post-transfection, the transfection complex-containing medium was replaced with fresh complete medium, and the cells were left to rest overnight before treatment.

### 2.9. Superoxide Measurement

Production of superoxide was assessed as previously described [41]. First, ATDC5 cells were grown on glass coverslips in a 12-well plate (8 × 10^4^ cells/well) for 24 h. Cells were incubated with increasing concentrations of either NaHS or P* (0.25–1 mM) for 1 h at 37 °C, washed, and further incubated with an incubation buffer containing 10 µM hydroethidine and increasing concentrations of NaHS or P* (0.25–1 mM) with menadione (100 µM) for 4 h at 37 °C. In another experiment, the ATDC5 cells were incubated with NaHS or P* (0.25–1 mM) for 24 h, washed, and incubated with an incubation buffer containing 10 µM hydroethidine and menadione (50 µM) for 4 h at 37 °C. Afterwards, fluorescence imaging was performed at room temperature using a Zeiss Axiovert 200 equipped with a xenon lamp, polychromator, Chroma filters (exciter: ET545/25; emitter: 605/70; beamsplitter: T565lp), and a CoolSNAP fx-HQ CCD-camera (Visitron Systems GmbH, Puchheim, Germany). For each condition, 10 images were taken randomly and analyzed using ImageJ (https://imagej.nih.gov/ij/, accessed in 2015). For background correction, fluorescence was measured in cell-free areas.

### 2.10. Quantification of IL-6

IL-6 levels in cell culture supernatants were quantified using a commercially murine IL-6 ELISA kit (Life Technologies, Thermo Fisher Scientific, Inc. Waltham, Vienna, Austria).

### 2.11. Quantitative Polymerase Chain Reaction (qPCR)

Total RNA was isolated using the AurumTM Total RNA Mini Kit (Bio-Rad Laboratories Ges.m.b.H., Vienna, Austria) according to the manufacturer protocols. Total RNA quality was determined by measuring OD260/OD280 by means of UV/Vis spectroscopy using a Nanodrop 2000 spectrophotometer (VWR International GmbH, Vienna, Austria). The cDNA synthesis was performed with the iScript cDNA Synthesis Kit (Bio-Rad Laboratories). The qPCR analysis was performed using TaqMan^®^ Universal PCR Master Mix and pre-designed TaqMan^®^ Gene Expression Assays (Thermo Fisher Scientific, Vienna, Austria) as follows: Nrf2 (Mm00477784_m1; accession number: NM_010902.3), IL-6 (Mm00446190_m1; accession number: NM_031168), Nrf2 (Mm00477784_m1; accession number: NM_01092.3), and cyclophilin D (Mm07298544_g1; accession number: NM_026352). Reactions were carried out using a StepOnePlusTM Real-Time PCR System (Thermo Fisher Scientific). Cycling conditions were as follows: 2 min 50 °C, 10 min 95 °C, 40 cycles of 15 s 95 °C, and 1 min 60 °C. Data were analyzed according to the 2^−∆∆Ct^ method using cyclophilin D as a reference gene.

In primary human chondrocytes from OA patients, isolation of total RNA, cDNA synthesis, and SYBR Green-based qPCR were conducted as previously described [40]. Succinate dehydrogenase A (SDHA) was used as a reference gene. The primers used were the following: SDHA (forward primer: 5′-TGGGAACAAGAGGGCATCTG-3′, reverse primer: 5′-CCACCACTGCATCAAATTCTAG-3′; accession number: NM_004168), IL-6 (forward primer: 5′-ATAGGACTGGAGATGTCTGAGG-3′, reverse primer: 5′-AGGCAACTGGACCAAGG-3′; accession number: NM_000600), MMP-13 (forward primer: 5′-TCAGGAAACCACCTCTCCAC-3′, reverse primer: 5′-TGACGCCAACAATACGGTTA-3′; accession number: NM_002427).

### 2.12. Statistical Analysis

All data are expressed as means ± SEM. Experiments were performed independently at least three times. Statistical analyses were performed with GraphPad Prism 9.0 (GraphPad Software, San Diego, CA, USA). If not otherwise stated, one-way ANOVA with Tukey’s post hoc test was used for statistical comparison. Only the statistical results for the highest concentrations used are shown in the figures for the purpose of clarity. Supplementary Table S1 includes all *p*-values. *p*-values less than 0.05 (*), 0.01 (**), 0.001 (***) or 0.0001 (****) were considered statistically significant.

## 3. Results

### 3.1. H_2_S Release and Cellular Uptake of P*

**Figure 2:**
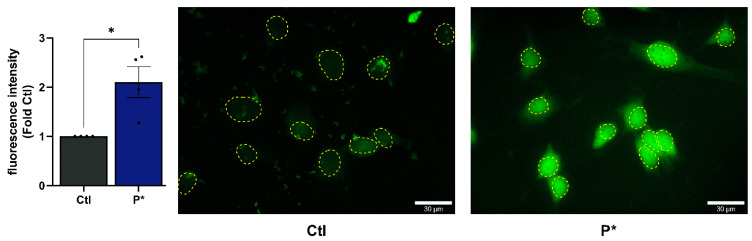
Intracellular H_2_S release by P*. ATDC5 cells were incubated for 30 min with the H_2_S fluorescent probe WSP-5 followed by incubation with 1 mM P* for 30 min. Approximate nuclear outlines (yellow) were traced from the corresponding bright-field images, which are presented in Supplementary Figure S2. Scale bar = 30 µm. Results are expressed as relative quantity compared to untreated cells (Ctl) set to 1, *n* = 4, * *p* < 0.05 (unpaired *t*-test).

P* only releases H_2_S in the presence of thiols, such as cysteine or glutathione, reaching the maximum level after approximately 10 min (Supplementary Figure S1). As free thiols are relatively abundant in mammals, we hypothesized that the intracellular amount of thiols would be sufficient to induce H_2_S generation from P*. This was verified by monitoring of the intracellular H_2_S release using the fluorescent probe WSP-5. Indeed, after incubation with 1 mM P* for 30 min, we observed a strong fluorescence signal (Figure 2, corresponding bright-field images are shown in Supplementary Figure S2) confirming the intracellular release of H_2_S by P* in the ATDC5 cells.

### 3.2. Cell Viability in Response to P* Administration

**Figure 3:**
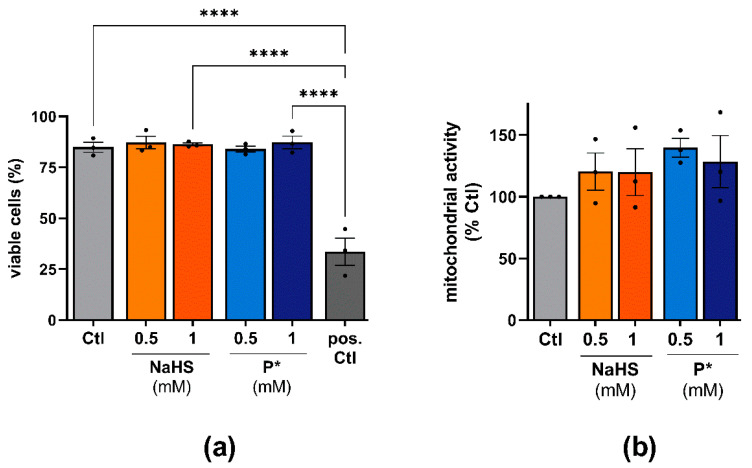
Effects of H_2_S donors on the cell viability of ATDC5 cells and primary human chondrocytes. (**a**) ATDC5 cells were incubated with 0.5 or 1 mM NaHS or P* for 24 h and cell viability was assessed using trypan blue staining. Untreated cells (Ctl) served as a negative control. Cells boiled for 5 min at 95 °C served as a positive control (pos. Ctl), *n* = 3, **** *p* < 0.0001. (**b**) Primary human chondrocytes were incubated for 24 h with 0.5 or 1 mM NaHS or P*. Cell viability was evaluated by MTT assay with Ctl set to 100%, *n* = 3.

To evaluate the effects of NaHS and P* on cell viability, we incubated ATDC5 or primary human OA chondrocyte cells for 24 h with NaHS or P*. In both cell types, NaHS and P* had no detrimental effects on cell viability in the concentrations used (Figure 3).

### 3.3. P* Activates the Nrf2 Pathway

**Figure 4:**
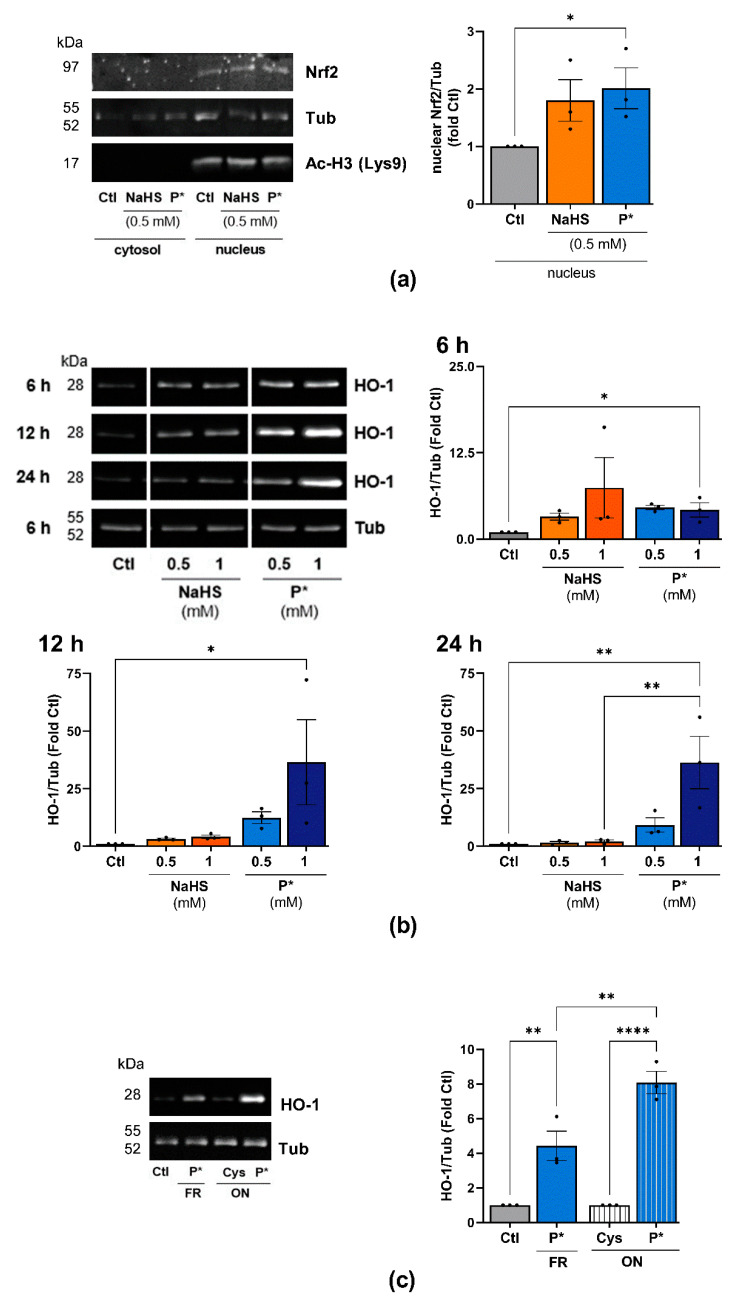
P* upregulates the Nrf2 pathway. (**a**) Western blot analysis of cytoplasmic and nuclear protein fractions in ATDC5 cells after 1 h of incubation with 0.5 mM NaHS or P*. Densitometry analysis values of Nrf2 (nuclear fraction) were normalized against α, β-tubulin (Tub) (nuclear fraction) and then compared with untreated cells (Ctl) set to 1, *n* = 3, * *p* < 0.05 (unpaired *t*-test). Acetyl-Histone H3 (Lysine 9, Ac-H3 (Lys9)) was used as a loading control for the nuclear proteins. (**b**) ATDC5 cells were incubated with increasing concentrations of NaHS or P* for 6, 12, or 24 h. Representative cropped Western blots of the expression of HO-1 and Tub. Full-size blots are presented in Supplementary Figure S3. Densitometry analysis values of HO-1 were normalized against Tub and then compared with untreated cells (Ctl) set to 1, *n* = 3. 6 h: ** *p* < 0.01 (unpaired *t*-test); 12 and 24 h: * *p* < 0.05, ** *p* < 0.01 (one-way ANOVA). (**c**) P* was decomposed by leaving solutions uncapped overnight (ON) at 37 °C in the presence of l-cysteine (1:1, ON P*). l-cysteine without P* served as control (ON Cys). The next day, ATDC5 cells were pre-incubated for 6 h with either 0.5 mM freshly prepared P* (FR) or decomposed P* (ON P*). Representative Western blots of HO-1 and Tub expression, *n* = 3, ** *p* < 0.01, **** *p* < 0.0001.

H_2_S was reported to activate Nrf2 via S-sulfhydration of Keap1, the negative regulator of Nrf2 [16,18]. Thus, Nrf2 dissociates from Keap1, allowing its translocation into the nucleus [42]. To assess this signaling cascade in ATDC5 cells, we determined the nuclear expression of Nrf2 after 1 h of incubation with 0.5 mM NaHS or P*. NaHS and P*, to a stronger extent, enhanced the nuclear translocation of Nrf2 (Figure 4a).

We further assessed the expression of the Nrf2 target gene HO-1 in response to NaHS and P* throughout the 24 h period. Both H_2_S donors strongly induced HO-1 expression compared to untreated cells (Figure 4b, uncropped blots are presented in Supplementary Figure S3). In the case of NaHS, HO-1 expression was maximal after 6 h of incubation and declined to control levels after 12 h. In contrast to NaHS, P*-induced HO-1 expression peaked at 12 h and was still highly elevated after 24 h of incubation.

Additionally, we assessed the expression of NQO1, another important target of Nrf2. After 6 or 12 h of incubation with NaHS or P*, we did not detect NQO1 protein (data not shown). After 24 h of incubation, both NaHS and P* induced NQO1 expression; again, the effect of P* appeared more pronounced (Supplementary Figure S4). As per- and polysulfides, rather than H_2_S, are likely responsible for Nrf2 activation via *S*-sulfhydration [42,43,44,45], we decomposed P* overnight into a mixture of tri- and tetrasulfides (as described in 2.1). First, equimolar concentrations of l-cysteine were added to enhance H_2_S release, while the formation of polysulfides takes place over time through condensation between P* and its generated persulfide. A detailed description of polysulfide formation from P* can be found in Supplementary Figure S5. The following day, we incubated ATDC5 cells for 6 h with either freshly prepared or decomposed P*. Decomposed P* induced HO-1 expression to a stronger extent than freshly prepared P* (Figure 4c).

### 3.4. P* Induces HO-1 via Activation of Nrf2 as Well as PI3K/Akt and p38 MAPK Pathways

**Figure 5:**
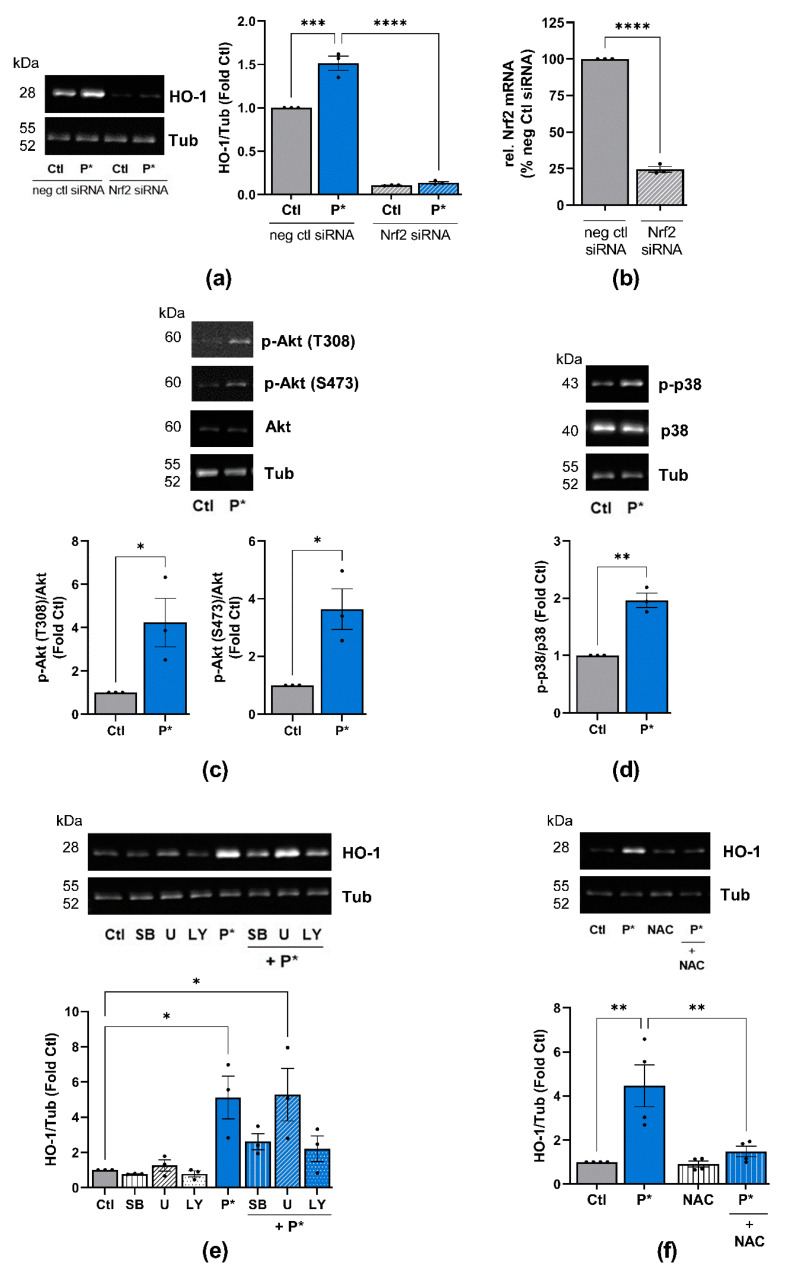
P*-mediated HO-1 induction requires activation of Nrf2, PI3K/Akt, and p38 MAPK pathways. (**a**) ATDC5 cells transfected with Nrf2 (Nrf2 siRNA) or negative control (neg ctl siRNA) siRNA were preincubated with 0.5 mM P* for 6 h. Expression of HO-1 was analyzed by Western blotting using α, β-tubulin (Tub) as control. Representative Western blots are shown. Densitometry analysis values of HO-1 were normalized against Tub and expressed as relative quantity compared to untreated cells (Ctl) set to 1, *n* = 3, *** *p* < 0.001, **** *p* < 0.0001. (**b**) Knockdown efficiency of Nrf2 by siRNA was validated by qPCR with cyclophilin D as a reference gene, *n* = 3, **** *p* < 0.0001. (**c**,**d**) ATDC5 cells were incubated with 0.5 mM P* for 6 h, and protein expression was analyzed through Western blotting. Representative Western blots of the expression of (**c**) p-Akt, Akt and Tub or (**d**) p-p38, p38 MAPK and Tub are shown. Densitometry analysis values of phosphorylated proteins were normalized against equivalent unphosphorylated proteins and then compared to untreated cells (Ctl) set to 1, *n* = 3, * *p* < 0.05, ** *p* < 0.01 (unpaired *t*-test). (**e**,**f**) Representative Western blots of the expression of HO-1 and Tub. Densitometry analysis values of HO-1 were normalized against Tub and expressed as relative quantities compared to Ctl set to 1, *n* = 3, * *p* < 0.05, ** *p* < 0.01. ATDC5 cells were incubated for 1 h with (**e**) p38 MAPK inhibitor SB203580 (SB, 10 µM), MEK1/2 inhibitor U0126 (U, 10 µM), or PI3K inhibitor LY294002 (LY, 10 µM), or (**f**) 2 mM N-acetylcysteine (NAC), followed by 6 h incubation with 0.5 mM P*.

To clarify whether activation of the Nrf2 signaling pathway was responsible for the induction of HO-1 by P*, we silenced Nrf2 through the transfection of ATDC5 cells with Nrf2 siRNA. The suppression of Nrf2 by siRNA abolished both basal and P*-induced HO-1 expression (Figure 5a). The knockdown efficiency of Nrf2 (75.5 ± 1.94%, *p*-value < 0.0001) was validated by qPCR (Figure 5b).

Apart from Nrf2, the PI3K/Akt, p38 MAPK and ERK1/2 pathways are considered to play central roles in the upregulation of HO-1 expression [46,47,48]. Moreover, we previously demonstrated that NaHS significantly activates Akt and ERK2 in unstimulated fibroblast-like synoviocytes from OA patients, while p38 MAPK is not affected [4]. Thus, we determined whether P* activates these pathways in ATDC5 cells. After 6 h of incubation with P*, the activation of Akt (p-Akt T308 and S473) and p38 (p-p38) MAPK was significantly augmented (Figure 5c,d), while ERK1/2 phosphorylation was not affected (Supplementary Figure S6). These findings suggest that the activation of PI3K/Akt and p38 MAPK pathways may be required for P*-induced HO-1 expression, in addition to Nrf2.

To confirm this assumption, we incubated ATDC5 cells for 1 h with inhibitors for p38 MAPK (SB203580), ERK1/2 (inhibitor of the upstream activator MEK1/2-U0126) and PI3K/Akt (LY294002), followed by incubation with P* for 6 h. Indeed, inhibition of p38 MAPK and PI3K/Akt, but not ERK1/2, clearly reduced P*-mediated HO-1 induction (Figure 5e). Since oxidative stress mainly activates p38 MAPK, we preincubated ATDC5 cells with the antioxidant N-acetyl cysteine (NAC) for 1 h followed by incubation with P* for 6 h. Indeed, NAC abolished P* mediated HO-1 expression (Figure 5f).

### 3.5. P* Exerts Antioxidative Effects

**Figure 6:**
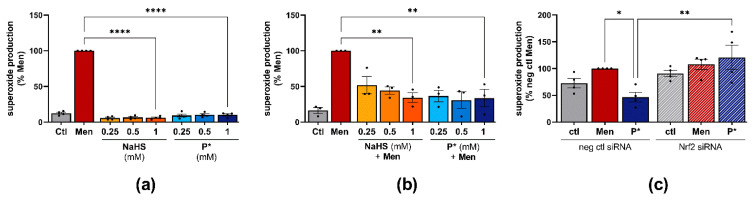
Antioxidative effects of P*. Ctl = untreated cells, representative fluorescence images of superoxide measurement are presented in Supplementary Figures S8 and S9. (**a**) Superoxide production in response to NaHS or P* was measured in ATDC5 cells and normalized to menadione (Men) set to 100%, *n* = 4, **** *p* < 0.0001. (**b**) Superoxide production in response to 24 h NaHS or P* followed by 4 h Men (50 µM), normalized to Men (100%). *n* = 3, ** *p* < 0.01. (**c**) ATDC5 cells transfected with Nrf2 (Nrf2 siRNA) or negative control (neg ctl siRNA) siRNA were preincubated with 1 mM P* for 24 h. After 4 h of incubation with Men (50 µM), superoxide production was measured and normalized to neg ctl Men set to 100%, *n* = 4, * *p* < 0.05, ** *p* < 0.01.

HO-1, as a stress-inducible enzyme, is generally induced by reactive oxygen species (ROS) [49]. Therefore, we measured the generation of superoxide by NaHS and P*. Menadione, which is an established inducer of oxidative stress, was used as a positive control. After 5 h of incubation, menadione excessively generated superoxide, whereas NaHS and P* did not (Figure 6a).

To clarify whether P* exerts antioxidative effects, we incubated ATDC5 cells with NaHS or P* for 24 h followed by medium exchange and incubation with menadione for 4 h. Both P* and NaHS significantly reduced menadione-induced superoxide production (Figure 6b). At the lowest concentration (0.25 mM), P* appeared more effective than NaHS. As the intrinsic HO-1 inducer hemin exhibited similar effects (Supplementary Figure S7), we determined whether P* is able to reduce menadione-induced superoxide production in Nrf2 silenced ATDC5 cells. Nrf2 silencing by transfection significantly abolished P*-mediated antioxidative effects (*p*-value = 0.028), (Figure 6c). Representative fluorescence images of all superoxide measurements are presented in Supplementary Figures S8 and S9.

### 3.6. Effects of P* on IL-6 Expression in ATDC5 Cells and Primary Human Chondrocytes

Apart from protecting cells against oxidative stress, HO-1 also exerts anti-inflammatory effects. Hence, we examined the effects of NaHS and P* on IL-6 expression in ATDC5 cells and in human OA chondrocytes.

**Figure 7:**
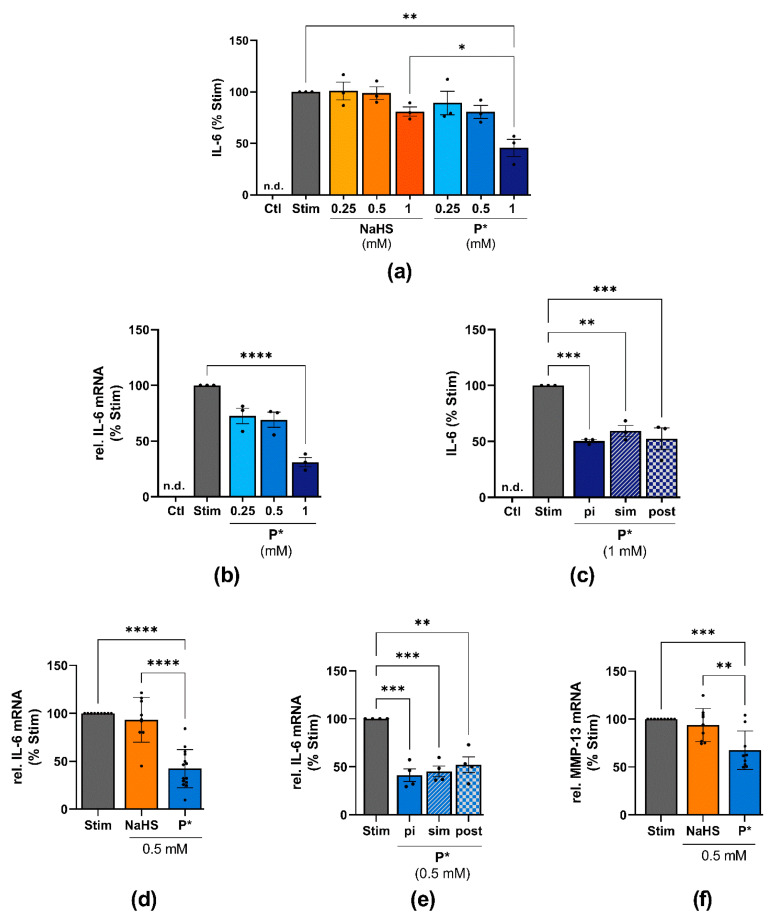
Effects of P* on IL-6 expression in ATDC5 cells and primary human chondrocytes. Ctl = untreated cells, n.d. = non-detected; significance is only shown for the highest concentrations used. (**a**) ATDC5 cells were treated with the indicated concentrations of NaHS or P* for 1 h followed by 24 h of stimulation with IL-1β/IFN-γ. IL-6 levels were assessed by ELISA and normalized to stimulated cells (Stim) set to 100%, *n* = 3, * *p* < 0.05, ** *p* < 0.01. (**b**) ATDC5 cells were incubated with P* for 1 h and stimulated for 24 h. IL-6 mRNA levels were determined by qPCR with cyclophilin D as a reference gene and normalized to Stim set to 100%, *n* = 3, **** *p* < 0.0001. (**c**) ATDC5 cells were incubated with 1 mM P* 1 h before (pi), simultaneously (sim), or 1 h after (post) 24 h of stimulation with IL-1β/IFN-γ. IL-6 levels were assessed by ELISA and normalized to Stim (100%), *n* = 3, ** *p* < 0.01, *** *p* < 0.001. (**d**) Primary human chondrocytes from OA patients were preincubated for 1 h with 0.5 mM NaHS or P* and stimulated for 24 h with IL-1β. IL-6 mRNA levels were determined by qPCR with SDHA as a reference gene and normalized to Stim (100%). NaHS: *n* = 9, P*: *n* = 14, **** *p* < 0.0001. (**e**) Primary human chondrocytes from OA patients were incubated with 0.5 mM P* 1 h pi, sim, or post 24 h of stimulation with IL-1β. mRNA levels were determined by qPCR with SDHA as a reference gene and normalized to Stim (100%), *n* = 4, ** *p* < 0.01, *** *p* < 0.001. (**f**) Primary human chondrocytes from OA patients were preincubated for 1 h with 0.5 mM NaHS or P* and stimulated for 24 h with IL-1β. MMP-13 mRNA levels were determined by qPCR with SDHA as a reference gene and normalized to Stim set to 100%, *n* = 9, ** *p* < 0.01, *** *p* < 0.001.

After 24 h of stimulation with IL-1β/IFN-γ, the ATDC5 cells secreted large amounts of IL-6 (Figure 7a). Both NaHS and P* decreased IL-6 production when the cells were incubated for 1 h with the H_2_S donors before stimulation (Figure 7a). At 1 mM, NaHS decreased IL-6 levels by approximately 20%, but this did not reach statistical significance (*p* = 0.54). In contrast, P* reduced IL-6 protein levels by approximately 55% (*p* < 0.01). In line with this finding, P* decreased IL-6 at the transcriptional level by approximately 70% (*p* < 0.0001), (Figure 7b). We further determined whether P* also decreased IL-6 levels when the inflamed status had already been initiated. Indeed, P* decreased IL-6 levels in ATDC5 cells regardless of whether P* had been added 1 h before (pi), simultaneously (sim), or 1 h after (post) cell stimulation (Figure 7c). In addition, we incubated the ATDC5 cells for 1 h with P* or penicillamine, the non-sulfane-sulfur-containing parental drug of P*, before the cells were stimulated for 24 h. In contrast to P*, penicillamine did not significantly decrease IL-6 production (Supplementary Figure S10a).

To further substantiate these findings and demonstrate the potential usefulness of P* for OA treatment, IL-6 expression was also measured in human OA chondrocytes. Almost identical results were obtained. Once again, P* proved to be much more effective than NaHS (Figure 7d). As observed with ATDC5 cells, P* reduced IL-6 regardless of the timepoint at which P* had been added (Figure 7e), while penicillamine had no effect (Supplementary Figure S10b). In addition, we observed a similar inhibitory potential of P* on MMP-13 mRNA levels in OA chondrocytes. As such, P* reduced MMP-13 by approximately 30% (*p* < 0.001), whereas NaHS did not affect MMP-13 expression (Figure 7f).

### 3.7. P*-Mediated HO-1 Induction and IL-6 Decline

**Figure 8:**
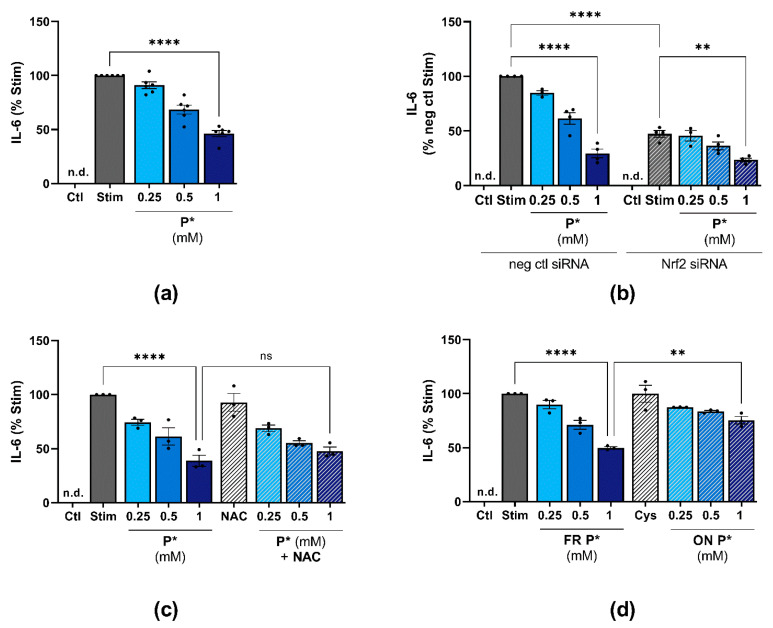
P*-mediated HO-1 induction and H_2_S release affect IL-1β/IFN-γ-induced IL-6 levels. IL-6 levels were determined by ELISA and normalized against stimulated cells (Stim) set to 100%. Ctl = untreated cells, n.d. = non-detected, ns = not significant. (**a**) ATDC5 cells were pre-incubated with indicated concentrations of P* for 6 h. Afterwards, P* was removed through medium exchange and cells were stimulated for 24 h with IL-1β/IFN-γ. *n* = 5, **** *p* < 0.0001. (**b**) ATDC5 cells transfected with Nrf2 (Nrf2 siRNA) or negative control (neg ctl siRNA) siRNA were preincubated with P* for 6 h, followed by a medium exchange and 24 h of stimulation with IL-1β/IFN-γ. *n* = 4, ** *p* < 0.01, **** *p* < 0.0001. (**c**) ATDC5 cells were treated with 2 mM N-acetylcysteine (NAC) for 1 h and subsequently with P* for 6 h. The medium was exchanged, and the cells were stimulated for 24 h with IL-1β/IFN-γ. *n* = 3, **** *p* < 0.0001. (**d**) P* was decomposed leaving solutions uncapped overnight (ON) at 37 °C in the presence of l-cysteine (1:1, ON Cys). The next day, ATDC5 cells were pre-incubated for 6 h with either freshly prepared P* or decomposed P* (ON P*) before the medium was exchanged followed by 24 h of stimulation with IL-1β/IFN-γ. *n* = 3, ** *p* < 0.01, **** *p* < 0.0001.

As HO-1 exerts anti-inflammatory effects, we elucidated the effect of P*-mediated HO-1 induction on IL-6 levels. First, we determined the effect of HO-1 induction before stimulating the cells with IL-1β/IFN-γ. For this purpose, we incubated ATDC5 cells for 6 h with P* (see Figure 3a), removed P* through medium exchange, and stimulated the cells with IL-1β/IFN-γ for 24 h. At 1 mM, P* still decreased IL-6 levels by approximately 55% (*p* < 0.01), (Figure 8a). This experimental setup was therefore used for all subsequent experiments.

To evaluate whether P* could decrease IL-6 levels in the absence of HO-1, we inhibited HO-1 induction by Nrf2 siRNA. Surprisingly, Nrf2 silencing significantly diminished IL-6 production (*p* < 0.0001) and P* further augmented the observed decline (*p* = 0.0012), (Figure 8b). In addition, co-incubation of P* with NAC did not significantly affect the P*-mediated IL-6 decrease (1 mM P*: 38.8 ± 5.1% vs. co-incubation with NAC: 47.8 ± 3.8%, *p* > 0.5) (Figure 8c), although in previous experiments, NAC had abolished P*-mediated HO-1 induction (see Figure 5f).

### 3.8. P* Decreases IL-6 Levels Dependent on H_2_S Release in ATDC5 Cells

We aimed to clarify whether the anti-inflammatory effects of P* were dependent on its immediate H_2_S release or mediated by the mixture of persulfides and polysulfides formed by the decomposition of P* [38]. Therefore, we compared the effects of freshly prepared P* with decomposed P* (preparation as described in 2.1) on IL-6 levels. Interestingly, while freshly prepared P* diminished IL-6 levels to 49.8 ± 1.1% (*p* < 0.0001), decomposed P* reduced IL-6 levels to only 74.4 ± 3.6% (*p* < 0.01), (Figure 8d).

### 3.9. P* Decreases IL-6 Levels Dependent on p38 MAPK Activation

**Figure 9:**
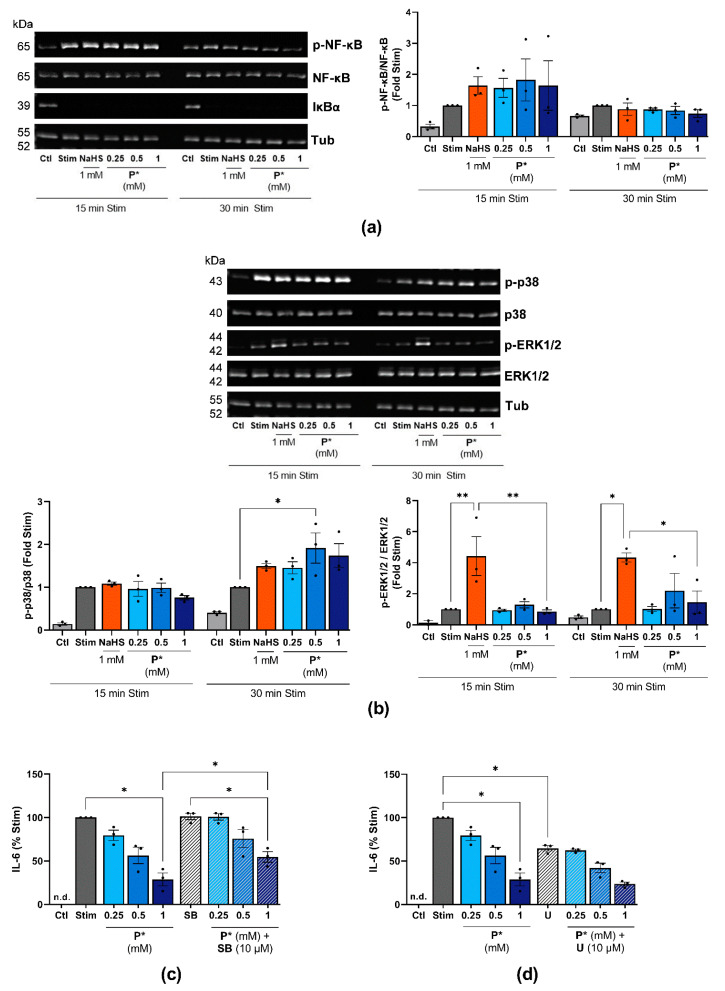
Influence of P* on NF-κB and MAPK signaling pathways. Ctl = untreated cells, n.d. = non-detected. (**a**,**b**) ATDC5 cells were incubated with NaHS or P* for 1 h and stimulated with IL-1β/IFN-γ for 15 or 30 min. Representative Western blots of (**a**) p-NF-κB, NF-κB, IκBα, and α, β-tubulin (Tub), or (**b**) p-p38, p38 MAPK, p-ERK1/2, ERK1/2, and Tub expression. Densitometry analysis values were normalized against (**a**) Tub or (**b**) the equivalent unphosphorylated protein, respectively, and then compared with stimulated cells (Stim) set to 1, *n* = 3. *p*-values for all concentrations are shown, * *p* < 0.05, ** *p* < 0.01. (**c**,**d**) ATDC5 cells were treated with (**c**)10 µM SB203580 (SB) or (**d**) 10 µM U0126 (U) for 1 h and subsequently with the indicated concentrations of P* for 6 h. The medium was exchanged, and the cells were stimulated for 24 h with IL-1β/IFN-γ. IL-6 levels were assessed by ELISA and normalized to Stim (100%), *n* = 3, * *p* < 0.05.

The NF-κB and MAPK signaling pathways are pivotal regulators of inflammation. Both pathways are also upregulated in OA, contributing to cartilage destruction [50]. Therefore, we examined whether P* decreases IL-6 levels via the inhibition of these pathways. We incubated ATDC5 cells for 1 h with NaHS or P* followed by 15 or 30 min stimulation with IL-1β/IFN-γ. Neither of the H_2_S donors blocked the activation of NF-κB p65 (p-NF-κB) or the degradation of IκBα, the negative regulator of NF-κB (Figure 9a). Presenting similar results, IL-1β-induced NF-κB activation was not affected by NaHS or P* in primary OA chondrocytes isolated from two patients (unpublished observation).

Regarding MAPKs, P* enhanced the IL-1β/IFN-γ-induced phosphorylation of p38 MAPK after 30 min of stimulation (Figure 9b). NaHS, but not P*, significantly increased the activation of ERK1/2 (Figure 9b). To clarify the possible interaction of P*, MAPK, and IL-6, we pretreated ATDC5 cells with the p38 MAPK inhibitor SB203580 or the MEK1/2 inhibitor U0126 for 1 h, followed by 6 h of treatment with P* and 24 h of stimulation. Surprisingly, inhibition of p38 MAPK activity by SB203550 did not alter IL-6 production, but it significantly diminished P*-induced decrease in IL-6 production (28.8 ± 7.4% vs. 54.6 ± 6.1% at 1 mM, *p* < 0.05), (Figure 9c). Blocking ERK1/2 activity using the MEK1/2 inhibitor U0126 decreased IL-6 production by approximately 33% (*p* < 0.05), while co-incubation with P* further enhanced this decrease by approximately 40% (*p* = 0.06), (Figure 9d). However, inhibition was of the same magnitude as it was in the control cells, strongly indicating that the ERK pathway was not involved in the P*-mediated inhibition of IL-6 production.

## 4. Discussion

**Figure 10:**
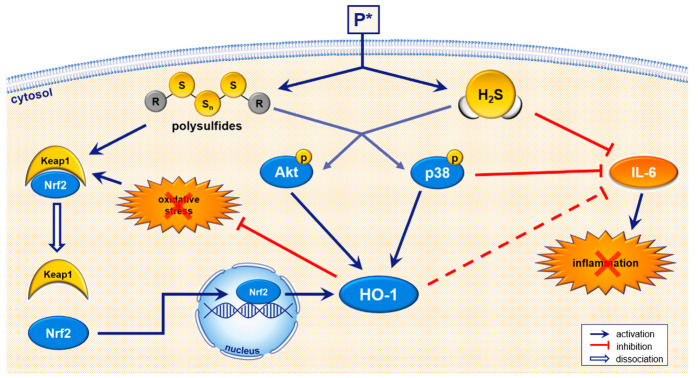
Schematic summary of P*-mediated biological effects. In aqueous buffers, P* generates a metastable persulfide and a mixture of polysulfides as end-products [38]. Upon entry into the cell, P* also releases H_2_S in the presence of thiols such as cysteine or glutathione. Sulfhydration of proteins is mainly carried out by polysulfides, leading to the activation of Nrf2/HO-1 dependent antioxidant pathways. P* also induces HO-1 via the regulation of Akt and p38 MAPK. Apart from antioxidant effects, P* also shows anti-inflammatory properties such as the inhibition of IL-6 for which H_2_S and p38 MAPK seem to be responsible.

In this study, we characterized the effects of the inducible and slow-releasing H_2_S and persulfide donor P* in the murine chondrocyte-like cell line ATDC5 on oxidative stress and inflammatory markers. We found that P* (i) induced the cytoprotective enzyme HO-1 via activation of Nrf2, PI3K/Akt and p38 MAPK pathways, (ii) exerted antioxidative effects via Nrf2 activation, and (iii) decreased IL-6 levels more efficiently than NaHS (summarized in Figure 10). Remarkably, to reduce oxidative stress, P* was primarily dependent on polysulfide formation. By contrast, H_2_S release was more important for P*-mediated IL-6 decrease. We further confirmed the anti-inflammatory effects of P* in primary human OA chondrocytes, a well-established model to study the basic mechanisms of OA pathobiology [51,52].

Although several studies showed beneficial aspects of H_2_S in OA [4,5,6,7,8], the data are controversial, and the pathways underlying the effects of H_2_S remain elusive. NaHS, albeit widely used, poorly reflects endogenous H_2_S production. When dissolved, it dissociates instantaneously, yielding high local H_2_S concentrations. Moreover, commercial NaHS often contains impurities that may be a major source of polysulfide formation in NaHS solutions [28,37,53]. Progress was achieved with the design of several slow-releasing H_2_S donors with GYY4137 as the best-known and most widely used representative [36,37]. GYY4137 releases H_2_S slowly upon hydrolysis, but the reaction kinetics as well as the resulting byproducts after H_2_S release have yet to be determined. These byproducts cannot be neglected, as sulfane sulfur species are reported to be important biologic mediators themselves [28,54,55]. Indeed, the resulting and hardly controllable formation of varying sulfur species with H_2_S donors may explain the diverse findings in response to H_2_S administration. To address these shortcomings, we used P* in this study: P* is water-soluble, stable at room temperature and releases H_2_S slowly and thiols-induced [38]. Furthermore, the formation of sulfur species is well characterized: at pH 7.4 in the absence of thiols, P* generates a meta-stable persulfide, quickly decomposing to polysulfides [38]. To differentiate between H_2_S and polysulfide-derived effects, P* can be decomposed in the presence of thiols thereby providing a tool to study sulfur species signaling under biologically relevant conditions.

Oxidative stress and ROS production have been reported to be elevated in OA, while antioxidant enzymes are decreased [56,57]. This imbalance results in excessive chondrocyte cell death. The transcription factor Nrf2 plays a key role in maintaining cellular redox homeostasis, and its negative regulator, Keap1, is an established target of S-sulfhydration [16,17,18,19]. Thus, we hypothesized that P* might exert antioxidant effects in ATDC5 cells through Nrf2 activation and the upregulation of its prominent targets HO-1 and NQO1. Generally, HO-1 synthesis is upregulated as a response to stress in biological systems. HO-1 serves as a potent endogenous antioxidant through the degradation of prooxidant heme to free iron, biliverdin, and CO, the latter of which also contributing to HO-1-mediated cytoprotection (e.g., [58,59]). Additionally, HO-1 exerts anti-inflammatory, anti-apoptotic, neuro-, cardio-, renal-, and hepatoprotective effects [27,49,60]. We found that treatment with both NaHS and P* induced HO-1 and the nuclear translocation of Nrf2. Additionally, menadione-mediated superoxide generation was abolished by NaHS and P*. Remarkably, P* proved to be substantially more effective than NaHS. While NaHS failed to induce HO-1 over a period of 12 h, HO-1 expression was still highly elevated after 24 h in response to P*. Nrf2 silencing by siRNA completely abrogated the P*-induced stimulation of HO-1 expression and the P*-mediated decline in menadione-induced superoxide production. Although the transfection itself increased the oxidative stress level demonstrated by the high basal level in negative control transfected ATDC5 cells, P* diminished menadione-induced superoxide. These data indicate that P* exerts antioxidant effects via the upregulation of the Nrf2 pathway and the ensuing HO-1 induction. Despite the numerous beneficial effects mediated by HO-1, HO-1 was also found to play a role in cancer progression by enhancing angiogenesis and inhibiting apoptosis [61,62,63,64,65]. Interestingly, while HO-1 promotes cancer progression, its activation is also essential in healthy cells to prevent carcinogenesis [66]. These versatile roles of HO-1 make it evident that care must be taken when therapeutically targeting HO-1 to avoid detrimental side effects.

S-Sulfhydration was described as the key signaling mechanism of H_2_S [14]. The reactive cysteines from Keap1, the negative regulator of Nrf2, can be S-sulfhydrated. Growing evidence indicates that S-sulfhydration is caused by per-/polysufides rather than by H_2_S [28,29,30,31]. Our results support this view, as decomposed P* induced HO-1 to a higher extent than freshly prepared P*. Additionally, a recent proteomic study identified 994 S-sulfhydrated proteins by NaHS [67], highlighting the importance of polysulfides as signaling molecules.

Apart from Nrf2 activation, the crosstalk between HO-1 upregulation and the PI3K/Akt, p38 MAPK, or ERK1/2 pathways is well-known [46,47,48]. H_2_S as well as polysulfides may affect these pathways to varying degrees [6,7,68], but only a few studies have investigated the possible interaction between the H_2_S/polysulfide-mediated activation of these pathways and HO-1 induction. We now provide evidence that P* induces HO-1 via activation of PI3K/Akt and p38 MAPK, but not ERK1/2. While P* increased Akt and p38 MAPK phosphorylation, specific inhibitors of these pathways significantly decreased P*-induced HO-1 expression. In agreement with our findings, Zhou et al. [69] and Shao et al. [70] observed that the activation of Nrf2 and HO-1 by NaHS was mediated by PI3K/Akt activation. Lohninger et al. [71] showed that the inhibition of p38 MAPK reversed HO-1 induction triggered by GYY4137 in THP-1 macrophages. These findings imply a crucial link between HO-1 induction by H_2_S donors and the MAPK and PI3K/Akt pathways. Interestingly, Zhang et al. [72] reported that NAC-polysulfides activated the Akt, p38 MAPK, and ERK1/2 pathways in RAW 264.7 cells. In concordance, Koike et al. [34] demonstrated that the polysulfide Na_2_S_4_ accelerated Akt phosphorylation and HO-1 expression in Neuro2A cells. Further studies are required to fully elucidate this crosstalk and to clarify the involvement of H_2_S and polysulfides in its regulation.

Accumulating evidence suggests that H_2_S plays an important role in inflammation [13,73,74]. To evaluate the anti-inflammatory properties of P* in comparison to NaHS, we used IL-6 production as a read-out because this cytokine is abundantly expressed in ATDC5 cells as well as in primary chondrocytes. In ATDC5 cells, P* significantly downregulated IL-1β/IFN-γ-induced IL-6 protein and mRNA expression, whereas the weak suppressive effect exerted by NaHS did not reach the level of significance. More importantly, P* also downregulated IL-6 levels when the inflamed status had already been initiated, i.e., after stimulation. In primary human chondrocytes from OA patients, we observed comparable inhibitory effects of P* on IL-6 and on MMP-13 expression. Once again, NaHS only weakly reduced the expression of the two markers.

Previous studies reported that several H_2_S donors exhibited anti-inflammatory effects via HO-1 induction in different OA models [75,76,77]. Considering the strong induction of HO-1 in response to P*, we studied the possible role of HO-1 in P*-mediated downregulation of IL-6. Inhibition of p38 MAPK abrogated P*-mediated HO-1 induction and IL-6 decrease indicating the importance of HO-1 for P* to decrease IL-6. When HO-1 induction was suppressed by silencing Nrf2 expression, the effects of P* on HO-1 expression were markedly reduced. However, silencing Nrf2 expression also diminished IL-6 levels significantl, y and P* further enhanced the observed decline. Similar to our results, Nrf2 activation was described to directly induce IL-6 transcription in hepatocytes [78] and persistent polyclonal B cell lymphocytosis B cells [79], indicating that Nrf2 suppresses pro-inflammatory markers dependent on the cell type and context. In addition, the antioxidant NAC significantly abolished P*-mediated HO-1 induction, but it did not affect the P*-mediated IL-6 decrease. Moreover, although decomposed P* induced HO-1 more efficiently than freshly prepared P*, it was significantly less efficient in reducing IL-6 expression. Taken together, these results demonstrate that in ATDC5 cells, HO-1 only plays a minor role for P* to diminish IL-6 levels. Our data further indicate that P* reduces IL-6 levels primarily via H_2_S release and not polysulfide formation, in contrast to its effect on HO-1 expression.

A major regulator of inflammation is NF-κB. Oh et al. [80] demonstrated that NaHS inhibited LPS-induced NF-κB activation in RAW 264.7 macrophages. Hence, we hypothesized that P* might downregulate IL-6 via NF-κB inhibition in ATDC5 or primary cells. However, neither NaHS nor P* blocked the phosphorylation of NF-κB or the degradation of IκBα. These results agree with previous findings of our group showing that NaHS does not inhibit NF-κB activation by IL-1β in C28/I2 chondrocytes [81]. In contrast to our data, NaHS was reported to reduce IL-1β-induced nuclear translocation [5] and activation of NF-κB [82] in articular chondrocytes from OA patients. These controversial findings are most likely explained by the differences in cell types and/or varying experimental conditions used in the studies.

In addition to NF-κB, the MAPK signaling pathways are abnormally activated in OA chondrocytes [50], and the inhibition of p38 MAPK was proposed as a therapeutic target in OA [83,84]. In our study, P* significantly enhanced p38 MAPK activation in ATDC5 cells, implying that P* administration might have adverse effects. However, when we inhibited p38 MAPK with the specific inhibitor SB203580, we did not observe an alteration of IL-1β/IFN-γ-induced IL-6 secretion, whereas P*-mediated downregulation of IL-6 was lessened. Moreover, P* required p38 MAPK activity to induce HO-1 expression. These results strongly indicate that both the suppressive and stimulatory effects of P* partially depend on p38 MAPK activation. It is becoming evident that p38 MAPK is a crucial factor in chondrocyte homeostasis: both the earlier stages (promotion of chondrogenesis) as well as the later stages (proliferation and hypertrophic differentiation of chondrocytes) of cartilage development require p38 MAPK signaling [85,86,87]. This might explain why the activation of p38 MAPK by P* in undifferentiated ATDC5 cells exerted beneficial effects, as they represent a chondroprogenitor cell line. At present, it is unknown whether P* also activates p38 MAPK in articular chondrocytes derived from healthy subjects or OA patients, which will be the subject of future research.

We chose to study the effects of P* at the cellular level in ATDC5 cells, as they provide a reliable chondrocyte-like cell culture model. The experiments in primary human OA chondrocytes corroborated the anti-inflammatory effects of P* observed in the cell line, supporting the validity of the ATDC5 model for this study. Furthermore, anti-inflammatory effects were also observed in synovial fibroblasts from OA patients (unpublished observation). Clearly, further studies are needed to fully understand the mechanism behind P*-mediated biological effects. In vivo administration of P* might further pave the way for the development of H_2_S/polysulfide donors as potential anti-inflammatory and antioxidant therapeutics in particular. However, the required concentrations of P* were relatively high and actually at the upper limit of pharmacologically acceptable doses. On the other hand, H_2_S concentrations in thermal waters used for the treatment of OA patients are quite comparable [88]. Therefore, the development of P* derived compounds with improved pharmacological properties might eventually lead to drugs that are suitable for therapeutic application in patients with musculoskeletal disorders and other chronic inflammatory conditions. Given the numerous beneficial effects of H_2_S and polysulfides, but also of Nrf2 and HO-1 on the regulation of the nervous and gastrointestinal system as well as cardiovascular and renal functions, P* might also protect against diseases affecting these organs.

## 5. Conclusions

In this study, we characterized the biological effects of P*, a H_2_S and persulfide donor. By decomposing P* into polysulfides, P* provides a valuable new research tool to study H_2_S and per-/polysulfide signaling in cells and tissues. Our work demonstrates the importance of not only H_2_S but also polysulfides as bioactive signaling molecules with potent antioxidant and anti-inflammatory effects.

## Data Availability

The authors declare that all data supporting the findings of this study are included in the article and/or the supplementary material. Additionally, raw data are available upon request from the corresponding author, B.K.

## Supplementary Materials

The following are available online at https://www.mdpi.com/article/10.3390/antiox10071049/s1, Figure S1: H_2_S generation by P*, Figure S2: Fluorescence and bright-field images, Figure S3: Original uncropped Western blot images related to Figure 4, Figure S4: P* induces NQO1 expression, Figure S5: Formation of tri- and tetrasulfides by P*, Figure S6: P* does not affect ERK1/2 phosphorylation, Figure S7: Hemin reduces menadione-induced superoxide production, Figure S8: Representative images of superoxide measurement, Figure S9: Representative images of superoxide measurement in transfected ATDC5 cells, Figure S10: Penicillamine does not affect IL-6 levels, Table S1: Summary of meaningful *p*-values.
