# Supplementary material for: Characterization of the Inducible and Slow-Releasing Hydrogen Sulfide and Persulfide Donor P*: Insights into Hydrogen Sulfide Signaling
Source: Antioxidants (Basel). 2021 Jun 29;10(7):1049. doi: 10.3390/antiox10071049 (PMC8300844; doi:10.3390/antiox10071049)
Supplement: Supplementary file 1 [file antioxidants-10-01049-s001.zip › antioxidants-1232571-supplementary.pdf]

Supplementary Material

	p-value	statistical analysis
**Fig. 2**		
Ctl vs. P* (1 mM)	0.0391	paired t-test
**Fig. 3, (a)**		
Ctl vs. NaHS (0.5 mM)	0.8594	
Ctl vs. NaHS (1 mM)	0.8694	
Ctl vs. P* (0.5 mM)	0.3741	
Ctl vs. P* (1 mM)	0.6623	
Ctl vs. P* (0.5 mM)	> 0,9999	
Ctl vs. P* (1 mM)	0,9961	
NaHS (1 mM) vs. P* (1 mM)	> 0,9999	
Ctl. vs. pos. Ctl	< 0,0001	
NaHS (1 mM) vs. pos. Ctl	< 0,0001	
P* (1 mM) vs. pos. Ctl	< 0,0001	
**Fig. 3, (b)**		
Ctl vs. NaHS (0.5 mM)	0.8594	
Ctl vs. NaHS (1 mM)	0.8694	
Ctl vs. P* (0.5 mM)	0.3741	
Ctl vs. P* (1 mM)	0.6623	
NaHS (1 mM) vs. P* (1 mM)	0.9932	
**Fig. 4, (a)**		
Ctl vs. NaHS (0.5 mM)	0,2090	
Ctl vs. P* (0.5 mM)	0,1095	
NaHS (0.5 mM) vs. P* (0.5 mM)	0,8702	
Ctl vs. NaHS (0.5 mM)	0.0907	unpaired t-test
Ctl vs. P* (0.5 mM)	0.0466	unpaired t-test
**Fig. 4, (b) 6 h**		
Ctl vs. NaHS (0.5 mM)	0.9310	
Ctl vs. NaHS (1 mM)	0.2405	
Ctl vs. P* (0.5 mM)	0.7272	
Ctl vs. P* (1 mM)	0.7913	
NaHS (1 mM) vs. P* (1 mM)	0.7942	
Ctl vs. NaHS (1 mM)	0.2159	unpaired t-test
Ctl vs. P* (1 mM)	0.0354	unpaired t-test
**Fig. 4, (b) 12 h**		
Ctl vs. NaHS (0.5 mM)	0.9990	
Ctl vs. NaHS (1 mM)	0.9961	
Ctl vs. P* (0.5 mM)	0.7391	
Ctl vs. P* (1 mM)	0.0412	
NaHS (1 mM) vs. P* (1 mM)	0.1161	
**Fig. 4, (b) 24 h**		
Ctl vs. NaHS (0.5 mM)	> 0.9999	
Ctl vs. NaHS (1 mM)	> 0.9999	
Ctl vs. P* (0.5 mM)	0.8799	
Ctl vs. P* (1 mM)	0.0027	
NaHS (1 mM) vs. P* (1 mM)	0.0035	
**Fig. 4, (c)**		

Ctl vs. FR P* (0.5 mM)	0.0083	
Ctl vs. ON Cys	> 0.9999	
Ctl vs. ON P* (0.5 mM)	< 0.0001	
ON Cys vs. ON P* (0.5 mM)	< 0.0001	
FR P* (0.5 mM) vs. ON P* (0.5 mM)	0.0055	
**Fig. 5, (a)**		
neg ctl siRNA: Ctl vs. P* (0.5 mM)	0.0001	
Nrf2 siRNA: Ctl vs. P* (0.5 mM)	0.9554	
neg ctl siRNA Ctl vs. Nrf2 siRNA Ctl	< 0.0001	
neg ctl siRNA P* (0.5 mM) vs. Nrf2 siRNA P* (0.5 mM)	< 0.0001	
**Fig. 5, (b)**		
neg ctl siRNA Ctl vs. Nrf2 siRNA Ctl	< 0.0001	unpaired t-test
**Fig. 5, (c)**		
p-Akt (T308): Ctl vs. P* (0.5 mM)	0.0450	unpaired t-test
p-Akt (S473): Ctl vs. P* (0.5 mM)	0.0204	unpaired t-test
**Fig. 5, (d)**		
Ctl vs. P* (0.5 mM)	0.0015	unpaired t-test
**Fig. 5, (e)**		
Ctl vs. SB	> 0.9999	
Ctl vs. U	> 0.9999	
Ctl vs. LY	> 0.9999	
Ctl vs. P* (0.5 mM)	0.0238	
Ctl vs. SB + P* (0.5 mM)	0.7949	
Ctl vs. U + P* (0.5 mM)	0.0179	
Ctl vs. LY + P* (0.5 mM)	0.9409	
P* (0.5 mM) vs. SB + P* (0.5 mM)	0.3274	
P* (0.5 mM) vs. U + P* (0.5 mM)	> 0.9999	
P* (0.5 mM) vs. LY + P* (0.5 mM)	0.1842	
**Fig. 5, (f)**		
Ctl vs. P* (0.5 mM)	0.0017	
Ctl vs. NAC	0.9993	
Ctl vs. NAC + P* (0.5 mM)	0.9025	
P* vs. NAC + P* (0.5 mM)	0.0053	
**Fig. 6, (a)**		
Ctl vs. Men	< 0.0001	
Ctl vs. NaHS (0.25 mM)	0.0400	
Ctl vs. NaHS (0.5 mM)	0.1003	
Ctl vs. NaHS (1 mM)	0.0620	
Ctl vs. P* (0.25 mM)	0.7254	
Ctl vs. P* (0.5 mM)	0.9454	
Ctl vs. P* (1 mM)	0.9215	
Men vs. NaHS (0.25 mM)	< 0.0001	
Men vs. NaHS (0.5 mM)	< 0.0001	
Men vs. NaHS (1 mM)	< 0.0001	
Men vs. P* (0.25 mM)	< 0.0001	
Men vs. P* (0.5 mM)	< 0.0001	
Men vs. P* (1 mM)	< 0.0001	
NaHS (1 mM) vs. P* (1 mM)	0.5007	
**Fig. 6, (b)**		

Ctl vs. Men	< 0.0001
Men vs. Men + NaHS (0.25 mM)	0.0235
Men vs. Men + NaHS (0.5 mM)	0.0074
Men vs. Men + NaHS (1 mM)	0.0017
Men vs. Men + P* (0.25 mM)	0.0024
Men vs. Men + P* (0.5 mM)	0.0010
Men vs. Men + P* (1 mM)	0.0015
Men + NaHS (1 mM) vs. Men + P* (1 mM)	> 0.9999
**Fig. 6, (c)**	
neg ctl siRNA: Ctl vs. Men	0,5599
neg ctl siRNA: Men vs. P* (1 mM)	0,0415
Nrf2 siRNA: Ctl vs. Men	0,8961
Nrf2 siRNA: Men vs. P* (1 mM)	0,9616
neg ctl siRNA P* (1 mM) vs. Nrf2 siRNA P* (1 mM)	0,0028
**Fig. 7, (a)**	
Stim vs. NaHS (0.25 mM)	> 0.9999
Stim vs. NaHS (0.5 mM)	> 0.9999
Stim vs. NaHS (1 mM)	0.5419
Stim vs. P* (0.25 mM)	0.9353
Stim vs. P* (0.5 mM)	0.5179
Stim vs. P* (1 mM)	0.0018
NaHS (1 mM) vs. P* (1 mM)	0.0496
**Fig. 7, (b)**	
Stim vs. P* (0.25 mM)	0.0266
Stim vs. P* (0.5 mM)	0.0146
Stim vs. P* (1 mM)	< 0.0001
**Fig. 7, (c)**	
Stim vs. P* (1 mM) pi	0.0004
Stim vs. P* (1 mM) sim	0.0030
Stim vs. P* (1 mM) post	0.0007
**Fig. 7, (d)**	
Stim vs. NaHS (0.5 mM)	0.7111
Stim vs. P* (0.5 mM)	< 0.0001
NaHS (0.5 mM) vs. P* (0.5 mM)	< 0.0001
**Fig. 7, (e)**	
Stim vs. P* (0.5 mM) pi	0.0003
Stim vs. P* (0.5 mM) sim	0.0006
Stim vs. P* (0.5 mM) post	0.0028
**Fig. 7, (f)**	
Stim vs. NaHS (0.5 mM)	0.6639
Stim vs. P* (0.5 mM)	0.0004
NaHS (0.5 mM) vs. P* (0.5 mM)	0.0035
**Fig. 8, (a)**	
Stim vs. P* (0.25 mM)	0.6812
Stim vs. P* (0.5 mM)	< 0.0001
Stim vs. P* (1 mM)	< 0.0001
**Fig. 8, (b)**	
neg ctl siRNA: Stim vs. P* (0.25 mM)	0,1181
neg ctl siRNA: Stim vs. P* (0.5 mM)	<0,0001

neg ctl siRNA: Stim vs. P* (1 mM)	<0,0001
Nrf2 siRNA: Stim vs. P* (0.25 mM)	>0,9999
Nrf2 siRNA: Stim vs. P* (0.5 mM)	0,3428
Nrf2 siRNA: Stim vs. P* (1 mM)	0,0012
neg ctl siRNA P* (1 mM) vs. Nrf2 siRNA P* (1 mM)	0,9055
**Fig. 8, (c)**	
Stim vs. P* (0.25 mM)	0.0324
Stim vs. P* (0.5 mM)	0.0008
Stim vs. P* (1 mM)	< 0.0001
Stim vs. NAC	0.9623
NAC vs. NAC + P* (0.25 mM)	0.0485
NAC vs. NAC + P* (0.5 mM)	0.0011
NAC vs. NAC + P* (1 mM)	0.0002
P* (1 mM) vs. NAC + P* (1 mM)	0.8847
**Fig. 8, (d)**	
Stim vs. FR P* (0.25 mM)	0.5526
Stim vs. FR P* (0.5 mM)	0.0009
Stim vs. FR P* (1 mM)	< 0.0001
Stim vs. Cys	> 0.9999
Cys vs. ON P* (0.25 mM)	0.3104
Cys vs. ON P* (0.5 mM)	0.0919
Cys vs. ON P* (1 mM)	0.0044
FR P* (1 mM) vs. ON P* (1 mM)	0.0030
**Fig. 9, (a)**	
15 min p-NF-κB/NF-κB	
Ctl vs. Stim	0.8953
Stim vs. NaHS (1 mM)	0.9146
Stim vs. P* (0.25 mM)	0.9466
Stim vs. P* (0.5 mM)	0.7999
Stim vs. P* (1 mM)	0.9130
NaHS (1 mM) vs. P* (1 mM)	> 0.9999
30 min p-NF-κB/NF-κB	
Ctl vs. Stim	0.3319
Stim vs. NaHS (1 mM)	0.9703
Stim vs. P* (0.25 mM)	0.9587
Stim vs. P* (0.5 mM)	0.9074
Stim vs. P* (1 mM)	0.5940
NaHS (1 mM) vs. P* (1 mM)	0.9444
**Fig. 9, (b)**	
15 min p-p38/p38	
Ctl vs. Stim	0.0002
Stim vs. NaHS (1 mM)	0.9793
Stim vs. P* (0.25 mM)	0.9996
Stim vs. P* (0.5 mM)	> 0.9999
Stim vs. P* (1 mM)	0.4226
NaHS (1 mM) vs. P* (1 mM)	0.1607
30 min p-p38/p38	
Ctl vs. Stim	0.1749
Stim vs. NaHS (1 mM)	0.3047
Stim vs. P* (0.25 mM)	0.3802
Stim vs. P* (0.5 mM)	0.0238
Stim vs. P* (1 mM)	0.0749

NaHS (1 mM) vs. P* (1 mM)	0.9469	
15 min p-ERK1/2 / ERK1/2		
Ctl vs. Stim	0.9084	
Stim vs. NaHS (1 mM)	0.0098	
Stim vs. P* (0.25 mM)	> 0.9999	
Stim vs. P* (0.5 mM)	0.9987	
Stim vs. P* (1 mM)	> 0.9999	
NaHS (1 mM) vs. P* (1 mM)	0.0073	
30 min p-ERK1/2 / ERK1/2		
Ctl vs. Stim	0.9844	
Stim vs. NaHS (1 mM)	0.0116	
Stim vs. P* (0.25 mM)	> 0.9999	
Stim vs. P* (0.5 mM)	0.6600	
Stim vs. P* (1 mM)	0.9904	
NaHS (1 mM) vs. P* (1 mM)	0.0311	
**Fig. 9, (c)**		
Stim vs. P* (0.25 mM)	0.2914	
Stim vs. P* (0.5 mM)	0.1803	
Stim vs. P* (1 mM)	0.0454	
Stim vs. SB	0.9998	
SB vs. SB + P* (0.25 mM)	0.8615	
SB vs. SB + P* (0.5 mM)	0.2507	
SB vs. SB + P* (1 mM)	0.0207	
P* (1 mM) vs. SB + P* (1 mM)	0.0112	
**Fig. 9, (d)**		
Stim vs. P* (0.25 mM)	0.2914	
Stim vs. P* (0.5 mM)	0.1803	
Stim vs. P* (1 mM)	0.0454	
Stim vs. U	0.0324	
U vs. U + P* (0.25 mM)	0.9933	
U vs. U + P* (0.5 mM)	0.3814	
U vs. U + P* (1 mM)	0.0627	
P* (1 mM) vs. U + P* (1 mM)	0.9294	
**Supplemental figure S4**		
Ctl vs. NaHS (0.5 mM)	0,9981	
Ctl vs. NaHS (1 mM)	0,9331	
Ctl vs. P* (0.5 mM)	0,0957	
Ctl vs. P* (1 mM)	0,2610	
NaHS (1 mM) vs. P* (1 mM)	0,6358	
Ctl vs. NaHS (1 mM)	0,0658	unpaired t-test
Ctl vs. P* (1 mM)	0,1223	unpaired t-test
**Supplemental figure S6**		
Ctl vs. P* (0.5 mM)	0,6130	unpaired t-test
**Supplemental figure S7**		
Ctl vs. Men	< 0,0001	
Men vs. Men + hemin (10 µM)	0,0005	
**Supplemental figure S10, (a)**		
Stim vs. P* (1 mM)	0,0016	
Stim vs. penicillamine (1 mM)	0,1041	
P* (1 mM) vs. penicillamine (1 mM)	0,0175	

Supplemental figure S10, (b)

Stim vs. P* (1 mM)	0,0016
Stim vs. penicillamine (1 mM)	0,0555
P* (1 mM) vs. penicillamine (1 mM)	0,0002

Supplemental Table S1. Summary of meaningful *p*-values. If not otherwise stated, one-way ANOVA with Tukey's posthoc test was used for statistical comparison.

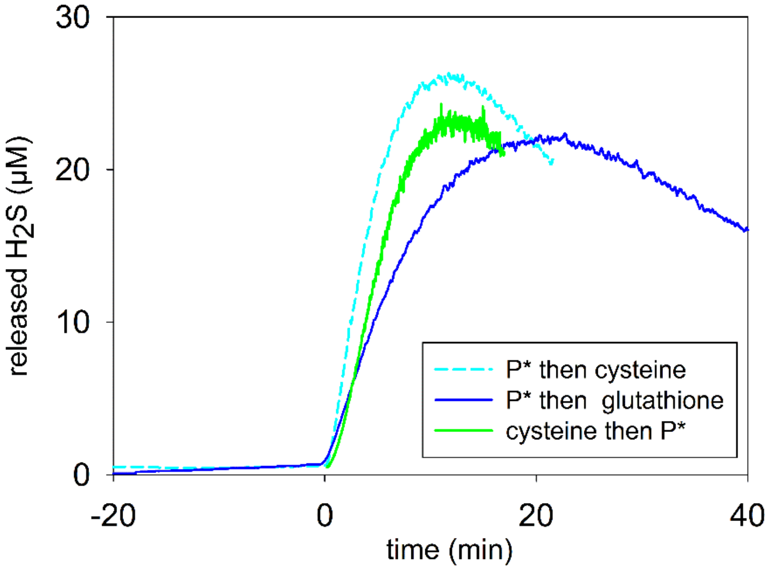

Supplemental figure S1. H_2S generation by P*. Thiol-induced H_2S release was monitored with the ISO-H_2S-2 sensor. A first substrate (P*, blue traces, or cysteine, green trace) was incubated in phosphate buffer (20 mM pH 7.4, containing 500 µM DTPA) for 20 minutes, then the second substrate (P*, cysteine or glutathione) was added (t = 0). Final concentrations were 40 µM for P* and 4 mM for L-cysteine or glutathione.

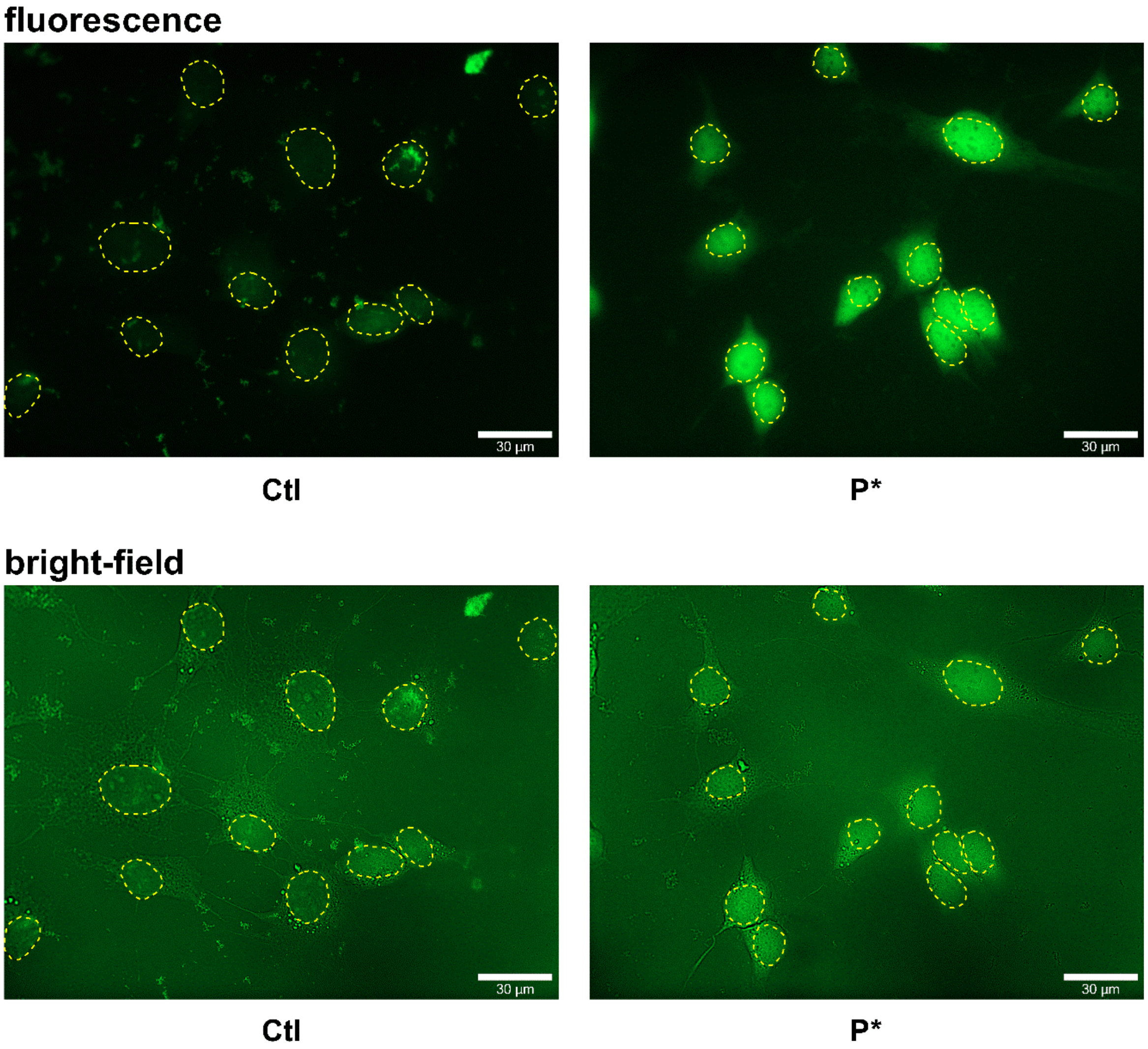

Supplemental figure S2. Fluorescence and bright-field images. ATDC5 cells were incubated for 30 min with the H_2S fluorescent probe WSP-5 followed by incubation with 1 mM P* for 30 min. Approximate nuclear outlines (yellow) were traced from the corresponding bright-field images presented above.

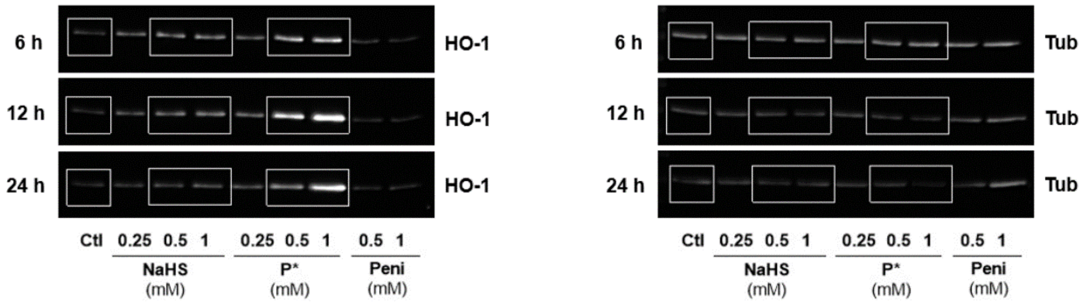

Supplemental figure S3. Original uncropped Western blot images related to Figure 4, P* induces HO-1 expression. ATDC5 cells were incubated with increasing concentrations of NaHS or P* for 6, 12 or 24 h. Representative full Western blots of the expression of HO-1 and α, β-tubulin (Tub).

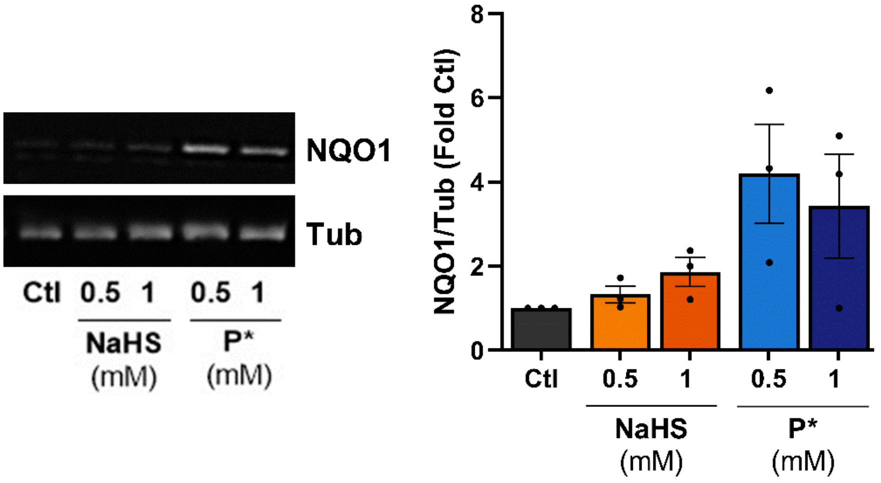

Supplemental figure S4. P* induces NQO1 expression. ATDC5 cells were incubated with increasing concentrations of NaHS or P* for 24 h. Representative Western blots of the expression of NQO1 and α, β-tubulin (Tub). Densitometry analysis values of NQO1 were normalized against Tub and then compared with untreated cells (Ctl) set to 1, n = 3.

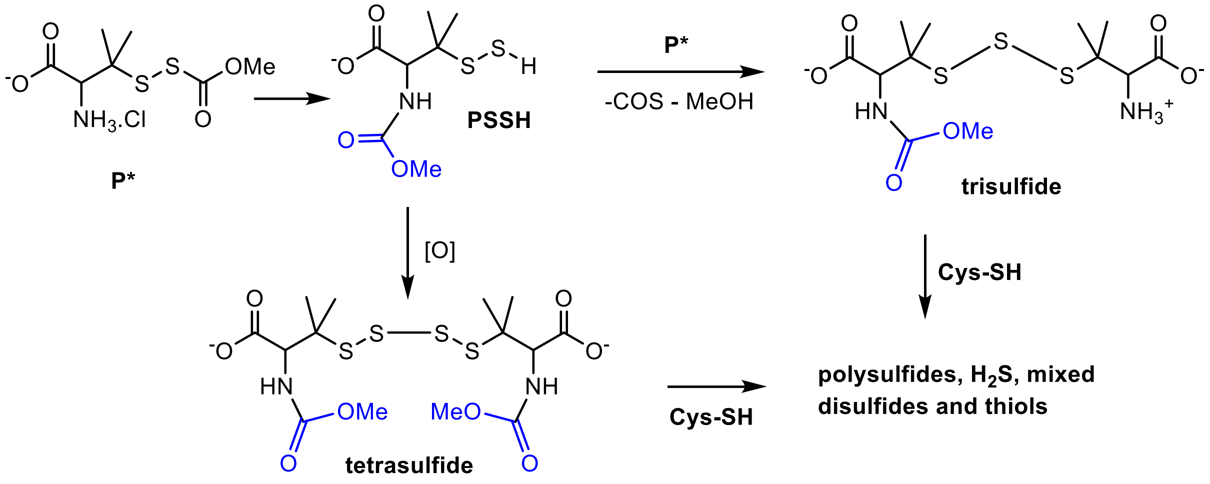

Supplemental figure S5. Formation of tri- and tetrasulfides by P*. P* is converted to its persulfide, PSSH, in a pH-driven S-to-N methoxycarbonyl transfer. P* may react with PSSH to give the trisulfide along with the volatiles COS and methanol, and PSSH can be oxidized to the tetrasulfide by traces of metal from the buffer. All these compounds may react with additional thiols to give complex dynamic mixtures of thiols, disulfides, polysulfides and hydrogen sulfide.

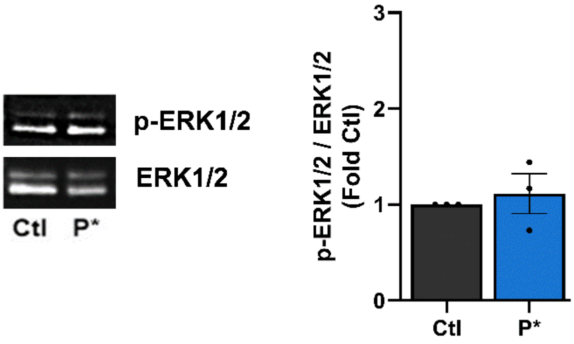

Supplemental figure S6. P* does not affect ERK1/2 phosphorylation. ATDC5 cells were incubated with 0.5 mM P* for 6 h. Representative Western blots of the expression of p-ERK1/2 and ERK1/2. Densitometry analysis values of p-ERK1/2 were normalized against ERK1/2 with respect to untreated cells (Ctl) set to 1, n = 3.

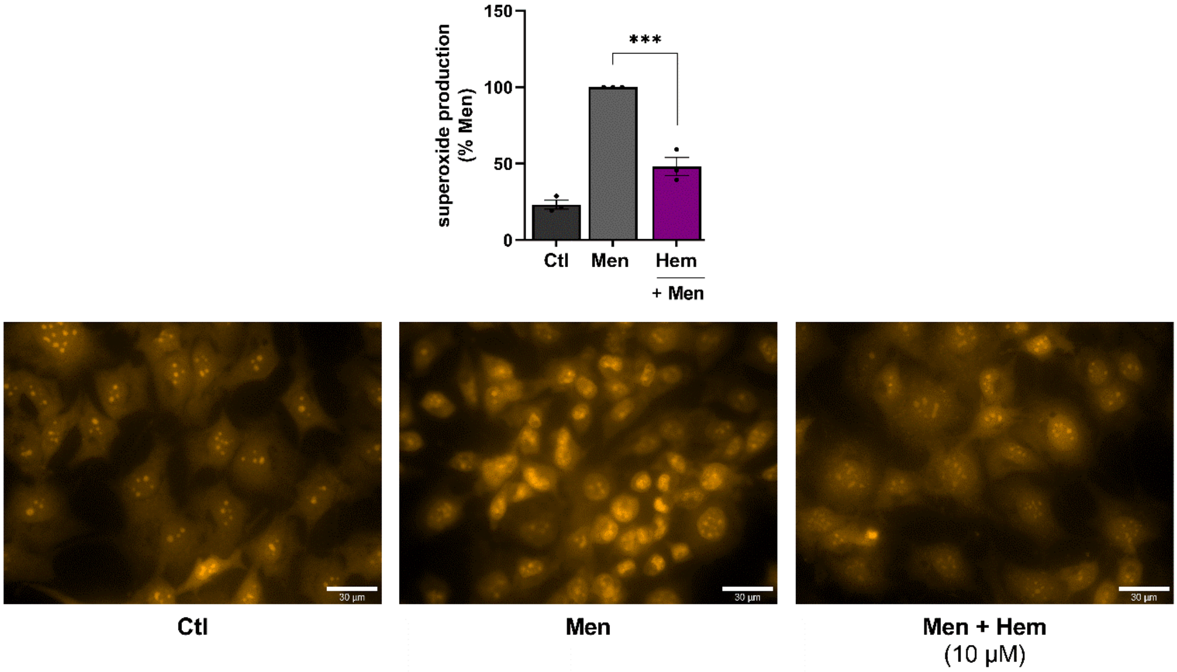

Supplemental figure S7. Hemin reduces menadione-induced superoxide production. ATDC5 cells were incubated with 10 µM hemin (Hem) for 24 h followed by 50 µM menadione (Men) for 4 h. Superoxide production was measured and normalized to Men (100 %), n = 3, *** $p < 0.001$.

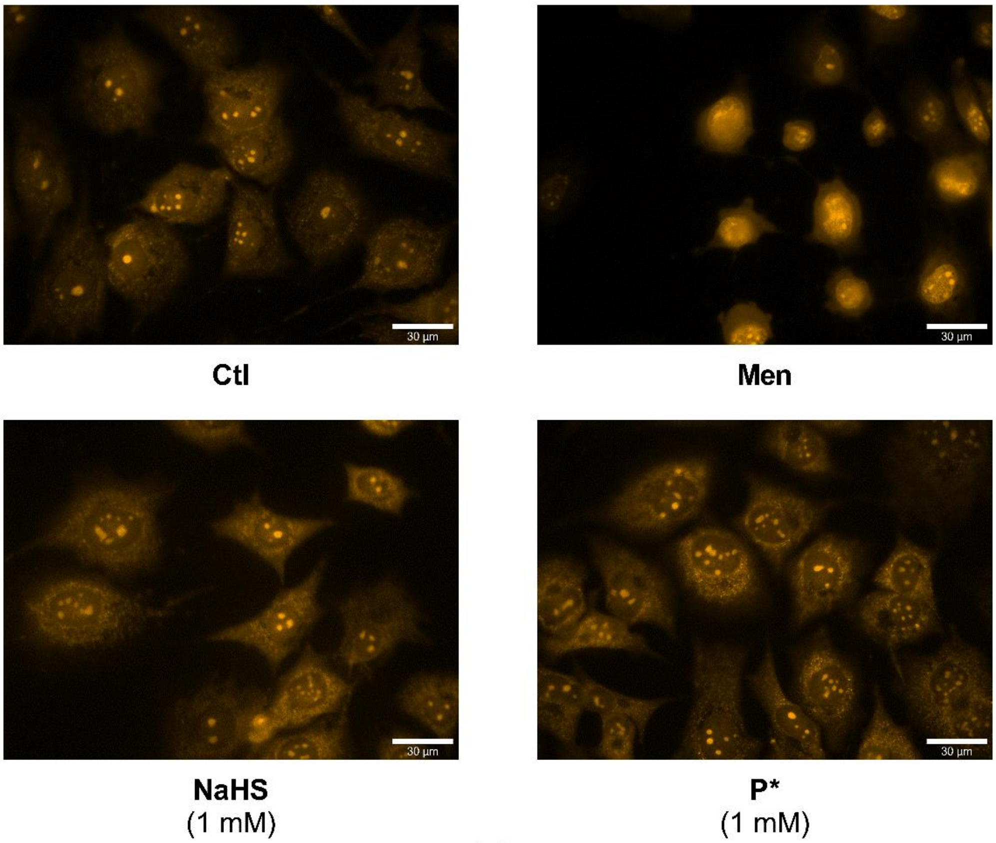

(a)

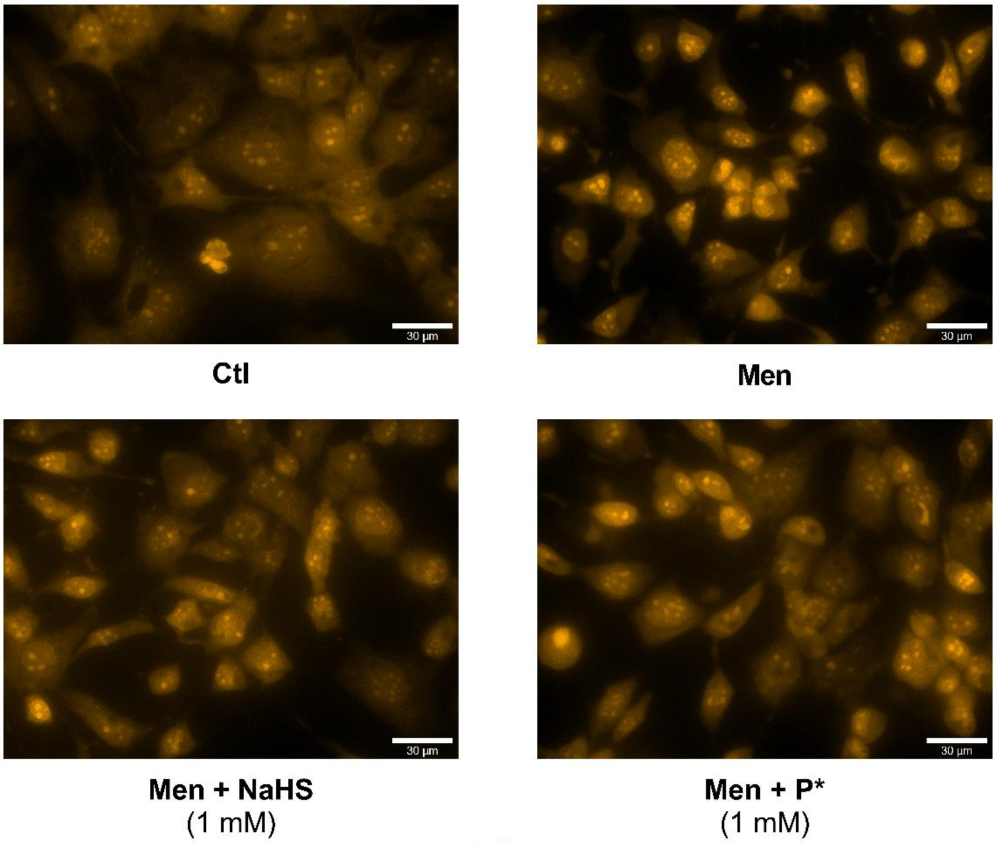

(b)

Supplemental figure S8. Representative images of superoxide measurement. (**a**) ATDC5 cells were incubated for 1 h with NaHS or P* followed by 4 h of incubation with 10 µM hydroethidine with or without 100 µM menadione (Men) before fluorescence imaging was performed. (**b**) ATDC5 cells were incubated for 24 h with NaHS or P*, washed and incubated with 10 µM hydroethidine with or without 100 µM menadione (Men) for 4 h before fluorescence imaging was performed.

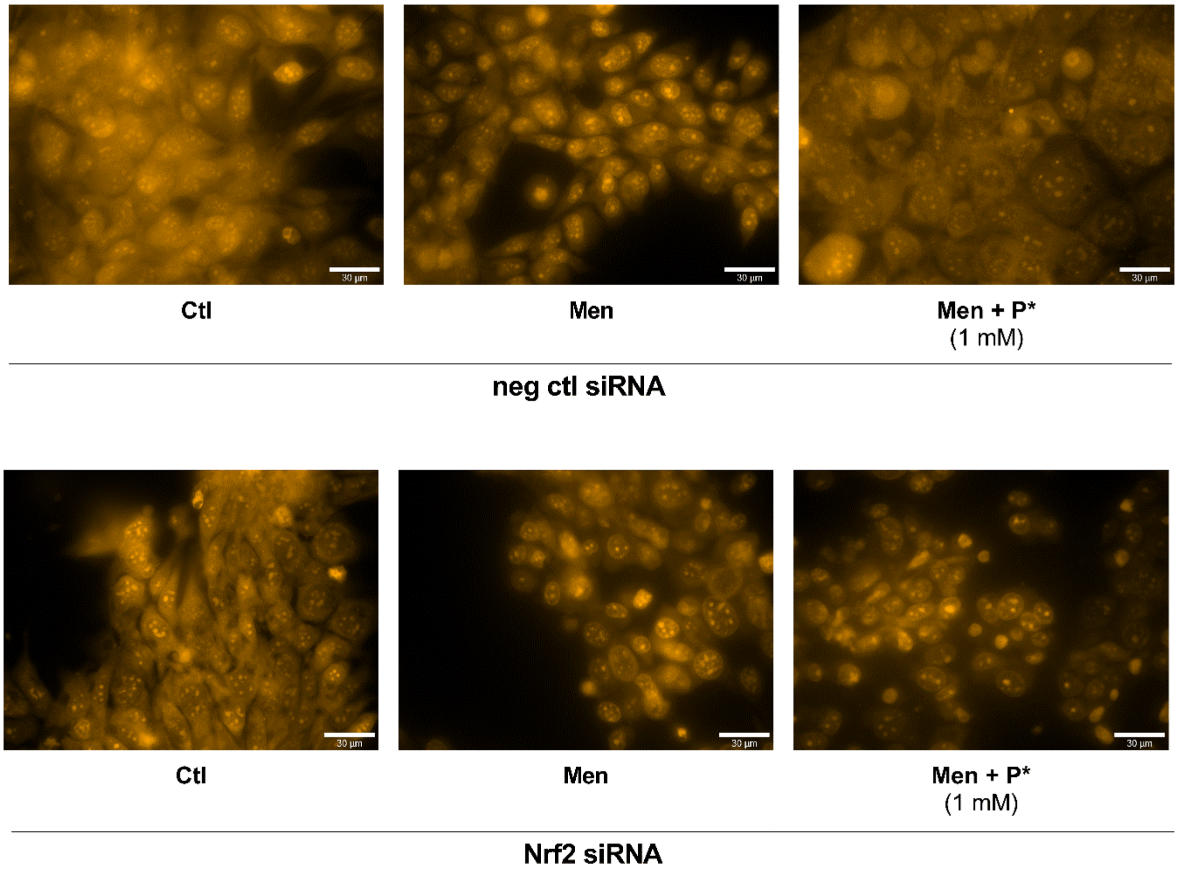

Supplemental figure S9. Representative images of superoxide measurement in transfected ATDC5 cells. ATDC5 cells transfected with Nrf2 (Nrf2 siRNA) or negative control (neg ctl siRNA) siRNA were incubated for 24 h with 1 mM P*, washed and incubated with 10 µM hydroethidine with or without 100 µM menadione (Men) for 4 h before fluorescence imaging was performed.

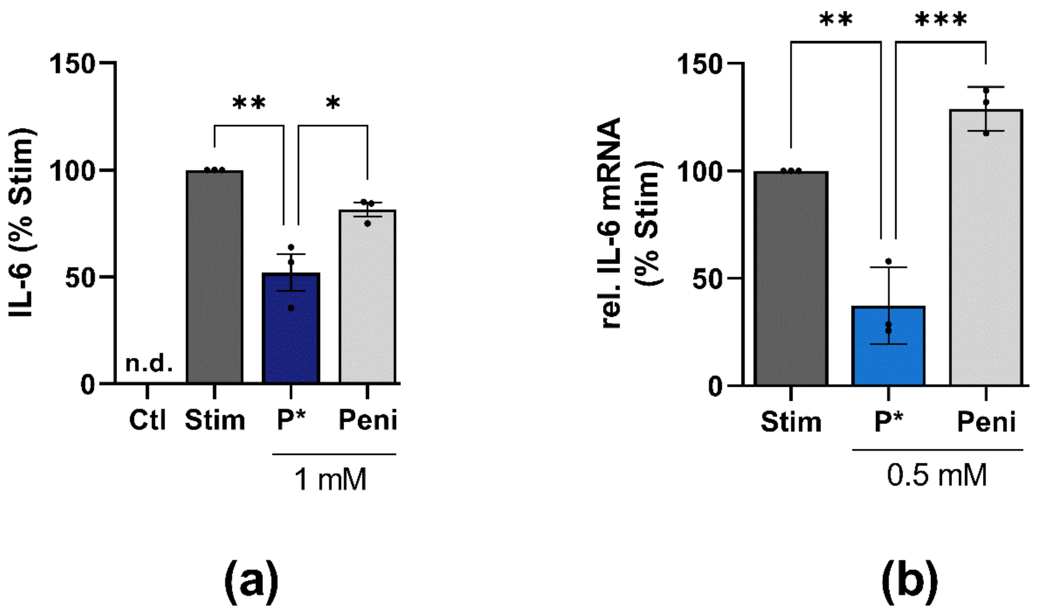

Supplemental figure S10. Penicillamine does not affect IL-6 levels. (**a**). ATDC5 cells were incubated with 1 mM P* or penicillamine (Peni) for 1 h followed by stimulation with IL-1β/IFN-γ for 24 h. IL-6 levels were determined by ELISA and normalized against stimulated cells (Stim) set to 100 %, n = 3, * $p < 0.05$, *** $p < 0.001$. (**b**) Primary human chondrocytes from OA patients were preincubated for 1 h with 0.5 mM P* or Peni and stimulated for 24 h with IL-1β. IL-6 mRNA levels were determined by qPCR with SDHA as a reference gene and normalized to Stim (100 %), n = 3, ** $p < 0.01$, *** $p < 0.001$.
